# Response dynamics of midbrain dopamine neurons and serotonin neurons to heroin, nicotine, cocaine, and MDMA

**DOI:** 10.1038/s41421-018-0060-z

**Published:** 2018-11-06

**Authors:** Chao Wei, Xiao Han, Danwei Weng, Qiru Feng, Xiangbing Qi, Jin Li, Minmin Luo

**Affiliations:** 1 School of Life Sciences, Peking University, Beijing, 100871 China; 2 Peking University-Tsinghua University-NIBS Graduate Program, Peking University, Beijing, 100081 China; 3 National Institute of Biological Sciences (NIBS), Beijing, 102206 China; 4 Beijing Key Laboratory of Neuropsychopharmacology, Beijing Institute of Pharmacology and Toxicology, Beijing, 100850 China; 5 School of Life Sciences, Tsinghua University, Beijing, 100084 China

## Abstract

Heroin, nicotine, cocaine, and MDMA are abused by billions of people. They are believed to target midbrain dopamine neurons and/or serotonin neurons, but their effects on the dynamic neuronal activity remain unclear in behaving states. By combining cell-type-specific fiber photometry of Ca^2+^ signals and intravenous drug infusion, here we show that these four drugs of abuse profoundly modulate the activity of mouse midbrain dopamine neurons and serotonin neurons with distinct potency and kinetics. Heroin strongly activates dopamine neurons, and only excites serotonin neurons at higher doses. Nicotine activates dopamine neurons in merely a few seconds, but produces minimal effects on serotonin neurons. Cocaine and MDMA cause long-lasting suppression of both dopamine neurons and serotonin neurons, although MDMA inhibits serotonin neurons more profoundly. Moreover, these inhibitory effects are mediated through the activity of dopamine and serotonin autoreceptors. These results suggest that the activity of dopamine neurons and that of serotonin neurons are more closely associated with the drug's reinforcing property and the drug's euphorigenic property, respectively. This study also shows that our methodology may facilitate further in-vivo interrogation of neural dynamics using animal models of drug addiction.

## Introduction

Drug abuse causes serious health and social problems. The United Nations World Drug Report estimates that a quarter of a billion world population uses at least one illegal drug in a year^1^. Opiates and cocaine represent two of the most addictive and devastating drugs of abuse^2^. MDMA is a popular recreational drug that often causes hallucination, cognitive defects, and post-drug depression^3^. Nicotine, though legal and widely consumed in most societies, is linked to a multitude of malignant diseases including lung cancer^4^.

Intensive research has revealed many insights into the neurobiological mechanisms underlying the effects of drugs of abuse. Peripherally administered drugs cross the blood–brain barrier and bind to their specific molecular targets, including μ-opioid receptor by heroin, nicotinic cholinergic receptors by nicotine, and dopamine and/or serotonin transporters by cocaine and MDMA^5,6^. It is well-established that drugs of abuse hijack the midbrain dopamine reward pathway, induce excessive release of dopamine and cause long-term adaptations of neural circuits, which finally leads to addiction^5,7^. Emerging evidence indicates that reward processing also intimately involves serotonin neurons in the dorsal raphe nucleus (DRN)^8–10^, the major source of serotonin in the forebrain. Although the role of the serotonin pathway in drug addiction is often ignored, the dysfunction of serotonin signaling pathways is implicated in increased vulnerability to cocaine abuse^11–13^ and potentiated seeking for opiate^14^.

Given the difficulty in vivo recording in a cell-type-specific manner, we still lack systematic data about how various drugs of abuse directly affect the activity of dopamine neurons and serotonin neurons in freely behaving animals. Although microdialysis studies have detected the release of dopamine and serotonin within minutes during drug administration^15,16^, this technique does not precisely monitor neuronal activity at sub-second temporal resolution. Fast-scan cyclic voltammetry (FSCV) has been developed to evaluate the sub-second volume transmission of dopamine^17–20^. However, FSCV in freely behaving animals faces forbiddingly demanding technical challenges. Similarly, recordings from slice preparations fail to reveal in vivo kinetics of neuronal activity challenged with different doses of drugs of abuse^21,22^. Again, electrophysiological recordings in vivo are difficult and often lack cell-type specificity and require anesthetic treatment^23,24^.

Here we studied how acute exposure of cocaine, MDMA, heroin, or nicotine modulates the neuronal activity of VTA dopamine neurons and DRN serotonin neurons by combining fiber photometry with intravenous drug infusion. Fiber photometry, together with the genetically-encoded Ca^2+^ indicator GCaMP6, has permitted the tracking of the activity dynamics of cell-type-specific neurons with sub-second temporal resolution^25–27^; these ease-of-use techniques are well adapted for freely moving transgenic mice during drug infusion. Our study revealed several interesting features of how four commonly abused drugs dynamically and differentially modulate the activity of dopamine neurons and serotonin neurons in freely behaving states.

## Results

### Heroin activates both dopamine neurons and serotonin neurons

**Fig. 1:**
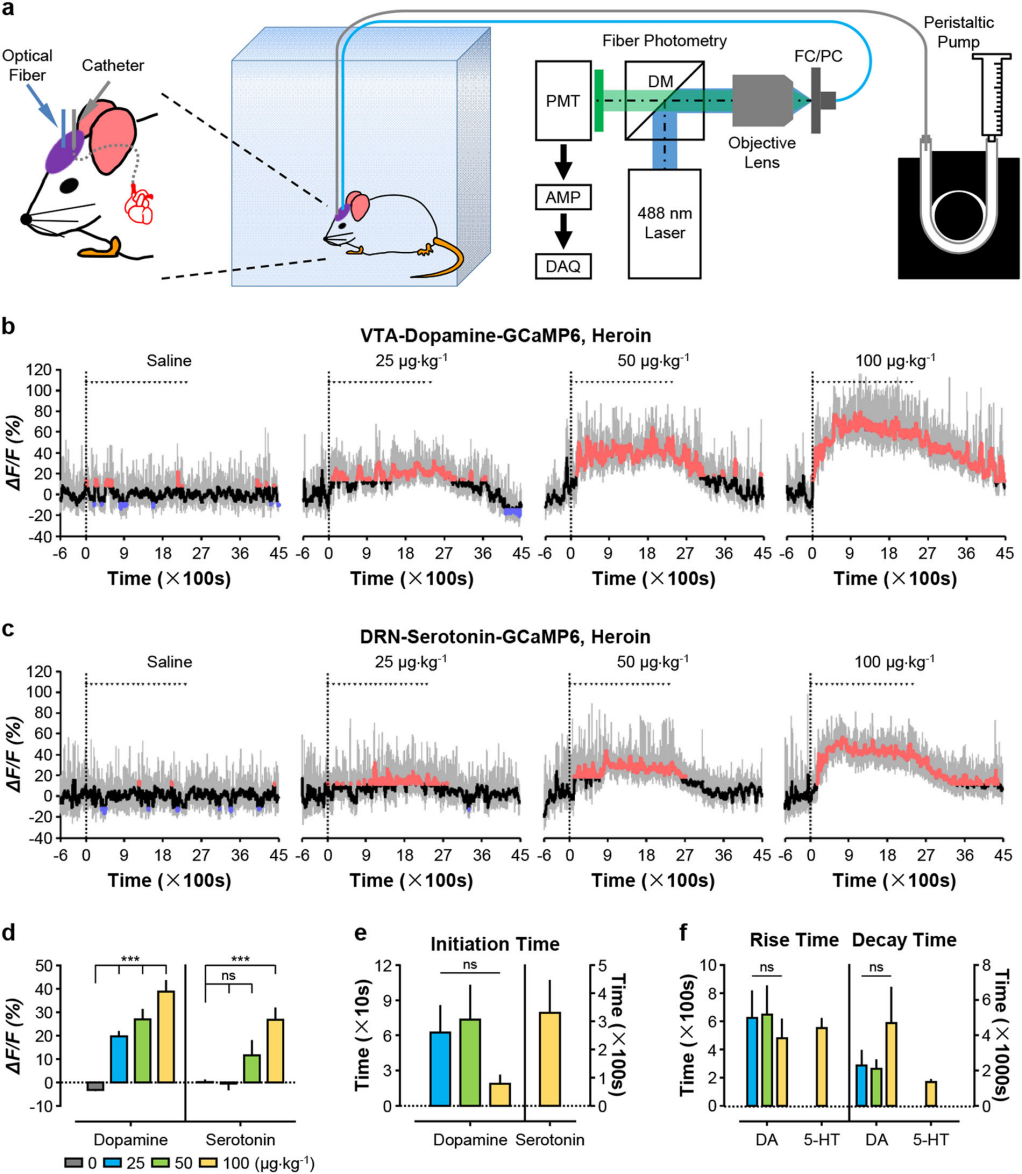
Acute heroin exposure causes long-lasting activation of both VTA dopamine neurons and DRN serotonin neurons in a dose-dependent manner. **a** Schematic of the experimental setup for fiber photometry of neuronal GCaMP fluorescence change (Ca^2+^ signals) from freely behaving mice that received intravenous drug infusions. **b**, **c** Ca^2+^ signals of dopamine neurons (**b**) and serotonin neurons (**c**) from two mice individually challenged with 20 intravenous infusions of heroin (small triangles) at the indicated doses. Small triangles indicate 20 repetitive infusions. The vertical dash line indicates the start of the first infusion. Gray, the original trace showing Ca^2+^ signals; black, the overall trend of Ca^2+^ signals. Moreover, red and blue indicate significant increase and decrease from the baseline before drug infusion, respectively (*p* < 0.05; permutation tests). **d** Overall Ca^2+^ signal intensities during the infusion phase [*n* = 6 dopamine mice; F(3, 20) = 24.47, *p* < 0.0001, one-way ANOVA with Tukey’s post-hoc test; *n* = 7 serotonin mice; F(3, 24) = 8.381, *p* = 0.0005, one-way ANOVA with Tukey’s post-hoc test]. **e** Initiation time required for heroin to activate dopamine neurons and serotonin neurons [*n* = 6 dopamine mice; F(2, 15) = 1.686, *p* = 0.2186, one-way ANOVA with Tukey’s post-hoc test]. **f** Rise time and decay time of the heroin-evoked Ca^2+^ signals [*n* = 6 dopamine mice, *n* = 7 serotonin mice; for rise time, F(2, 15) = 0.2508, *p* = 0.7814, one-way ANOVA with Tukey’s post-hoc test; for decay time, F(2, 15) = 1.180, *p* = 0.3342, one-way ANOVA with Tukey’s post-hoc test]. Error bars indicate SEM (**d**–**f**). *p < 0.05; **p < 0.01; ***p *<* 0.001; ns not significant, DM dichroic mirror, PMT photomultiplier tube, Amp amplifier, DAQ data acquisition interface

We combined an intravenous infusion system with fiber photometry to monitor real-time changes in neuronal activity from freely behaving mice challenged with drugs of abuse (Fig. 1a). Specifically, we targeted GCaMP6m to VTA dopamine neurons and DRN serotonin neurons by stereotaxically injecting Cre-dependent AAVs into the VTA of *DAT-Cre* mice (DAT-VTA-GCaMP6 mice for simplicity) or into the DRN of *Sert-Cre* (SERT-DRN-GCaMP6) mice (Supplementary Fig. S1a). During the surgical procedure, optical fiber tips were implanted at the injection sites (Supplementary Fig. S1b). Consistent with our previous studies^9,10,28^, GCaMP6m was selectively expressed within VTA dopamine neurons and within DRN serotonin neurons (Supplementary Fig. S1c).

After 1-week recovery for mice, we performed catheterization of right jugular vein, and 2 days later performed intravenous drug infusion. To mimic the drug intake patterns in self-administration studies, we made 20 infusions of the drug with the interval of 120 s between two consecutive infusions. We chose the doses commonly used to induce drug reinforcement (see methods for dosage concentrations and infusion parameters). We then performed fiber photometry to monitor changes in GCaMP fluorescence in response to drug infusions (Fig. 1b–d and Supplementary Fig. S1d) with two control experiments: saline infusion into GCaMP-expressing mice and drug infusion into GFP-expressing mice. No clear changes in green fluorescence occurred during GFP control experiments, thus ruling out that movement artefacts are responsible for change in GCaMP fluorescence (Supplementary Fig. S1e and S1f). For clarity, we refer to drug-induced changes in GCaMP fluorescence levels as “Ca^2+^ signals”.

We first examined the effects of heroin on neuronal activity. The three doses (0.025, 0.05, and 0.1 mg kg^−1^ per infusion) were chosen according to rodent self-administration studies, referring to the low, median and high doses^29^. Unlike natural rewards (including sucrose solution) that potently induce the phasic activation of both types of neurons^9,10^, acute heroin exposure significantly increased the Ca^2+^ signals of both VTA dopamine neurons and DRN serotonin neurons in a dose-dependent manner for up to an hour (Fig. 1b–d; Supplementary Fig. S2). For dopamine neurons, heroin caused ~20 to ~40% increase in Ca^2+^ signals (Fig. 1d). For serotonin neurons, heroin at the median and high doses (0.05 mg kg^−1^ and 0.1 mg kg^−1^) significantly increased Ca^2+^ signals by ~11% and ~26% with no significant effect at the low dose (0.025 mg kg^−1^) (Fig. 1d). Analyzing locomotor activity revealed that heroin challenge caused hyperactive locomotor activity in the time course that matched well with the increase in Ca^2+^ signals of both dopamine neurons and serotonin neurons (Supplementary Fig. S3).

We analyzed the kinetics of heroin-induced neuron activation. We calculated the initiation time by determining the time point after the first infusion when changes in Ca^2+^ signals became statistically significant. For dopamine neurons, the initiation time was ~60 s at the low dose, and was shortened to <20 s at the high dose (Fig. 1e), indicating that a single infusion significantly activated these neurons. The latency to the peak of Ca^2+^ signals ranged from 8 to 10 min, suggesting that the peak responses arrived following ~4 infusions (Fig. 1f). After the final infusion, Ca^2+^ signals decayed very slowly, and needed 40–80 min to return to the baseline (Fig. 1f). For serotonin neurons, at high dose, it required ~5.5 min to initiate a significant response and ~9 min to reach the peak of Ca^2+^ signals (Fig. 1e, f). Following the last infusion, Ca^2+^ signals slowly returned to the baseline for ~30 min (Fig. 1f).

### Nicotine activates dopamine neurons and produces biphasic responses

**Fig. 2:**
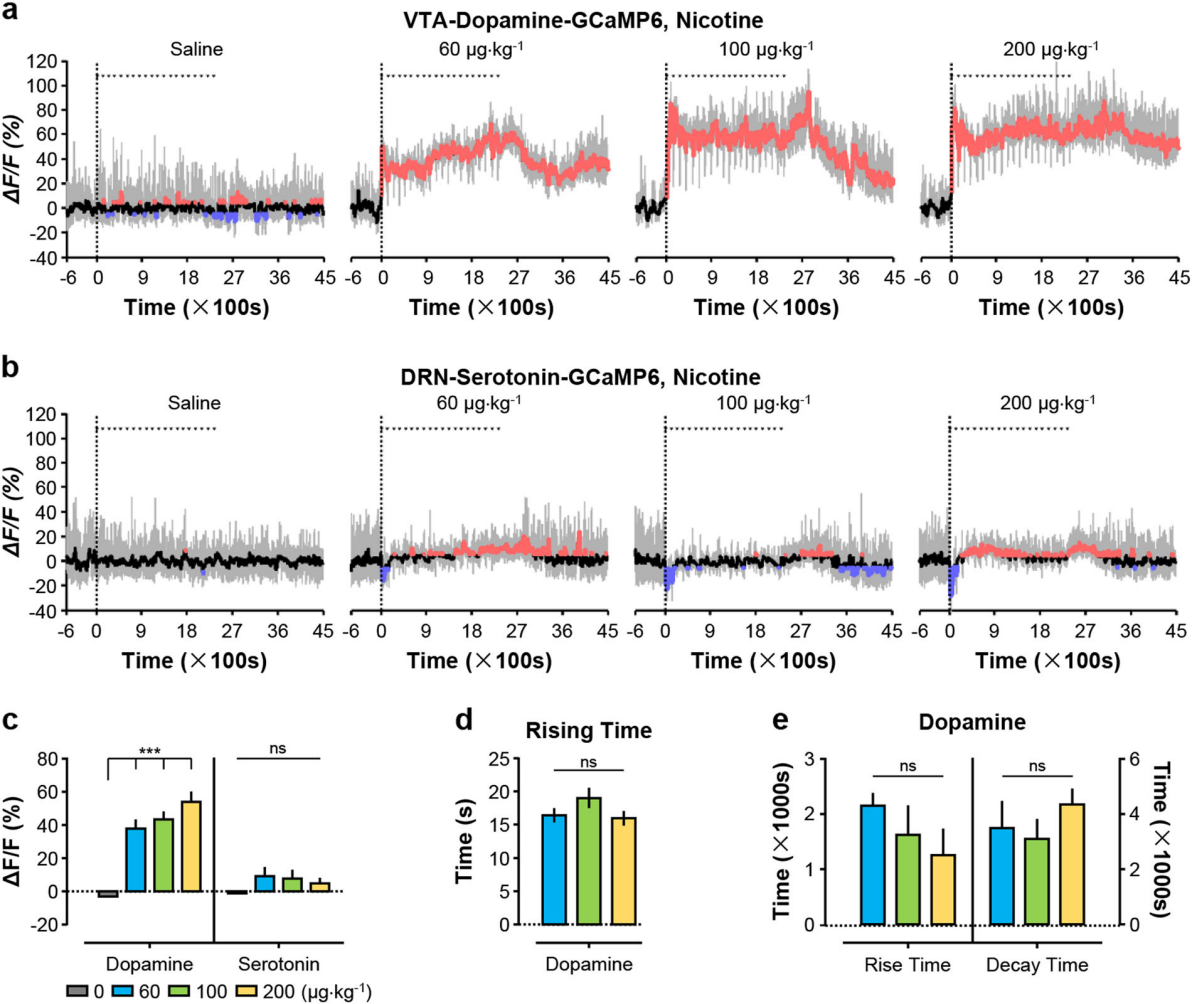
Nicotine rapidly and strongly activates VTA dopamine neurons but has only modest effect on DRN serotonin neurons. **a**, **b** Representative traces of Ca^2+^ signals from dopamine neurons and serotonin neurons treated with 20 infusions of nicotine at the indicated doses. **c** Overall Ca^2+^ signal intensities during nicotine infusions [*n* = 6 dopamine mice, F(3, 20) = 27.37, *p* < 0.0001, one-way ANOVA with Tukey’s post-hoc test; *n* = 7 serotonin mice, F(3, 24) = 1.235, *p* = 0.3188, one-way ANOVA with Tukey’s post-hoc test]. **d** The initiation time of Ca^2+^ signals following the first infusion of nicotine for VTA dopamine neurons [*n* = 6 dopamine mice; F(2, 15) = 1.013, *p* = 0.3866, one-way ANOVA with Tukey’s post-hoc test]. **e** Rise time and decay time of nicotine-evoked Ca^2+^ signals for VTA dopamine neurons [*n* = 6 dopamine mice; for rise time F(2, 15) = 1.050, *p* = 0.3742, one-way ANOVA with Tukey’s post-hoc test; for decay time, F(2, 12) = 0.6585, *p* = 0.5354, one-way ANOVA with Tukey’s post-hoc test]. Error bars indicate SEM (**c**–**e**). *p < 0.05; **p < 0.01; ***p < 0.001; ns not significant

We next investigated how VTA dopamine neurons and DRN serotonin neurons respond to nicotine. We tested three doses: 0.06, 0.1, and 0.2 mg kg^−1^ per infusion corresponding to the low, median, and high doses in rodent self-administration study^30^. At all three doses, the first infusion of nicotine alone was sufficient to cause a rapid increase in Ca^2+^ signals of dopamine neurons which continued to be elevated during the remaining infusions (Fig. 2a). At the high dose, nicotine infusions produce ~55% increases of Ca^2+^ signals (Fig. 2c). At a finer time scale, nicotine infusions in vivo biphasically modulated the Ca^2+^ signals in VTA dopamine neurons (Supplementary Fig. S4a). Although the initial infusion induced purely a rapid activation (Supplementary Fig. S4b), each subsequent infusion (infusions 2 through 20) caused an inhibition-then-rebound pattern: the Ca^2+^ signals was briefly suppressed and then quickly returned to the elevated level (Supplementary Fig. S4c). Unlike heroin, nicotine at the high dose caused a decrease in mouse locomotor activity that was correlated with nicotine-induced rapid increase in Ca^2+^ signal of dopamine neurons (Supplementary Fig. S5a and S5b).

For VTA dopamine neurons, kinetics analysis revealed that intravenous infusion almost instantly increased Ca^2+^ signals (initiation time: 2.2–4.4 s following the initial infusion at various doses) (Fig. 2d). The responses peaked within 16–19 s (Fig. 2e), making nicotine the fastest agent among the four drugs tested in this study. Once the final 20th infusion was completed, the Ca^2+^ signals reduced slowly, with a decay time of 51–72 min (Fig. 2e).

Nicotine exposure also increased the frequency of transient Ca^2+^ fluctuations from VTA dopamine neurons with no significant effects on the amplitude of transient events (Supplementary Fig. S6a, S6c and S6e). These transient fluctuations, which we henceforth refer to as “Ca^2+^ transients”, likely reflect neuronal activity at population level while mice explored surrounding environment.

In contrast to dopamine neurons, the overall effect of nicotine on serotonin neurons were very modest at all three dose (Fig. 2b, c). Furthermore, serotonin neurons showed decreases in the amplitude and frequency of Ca^2+^ transients during nicotine exposure (Supplementary Fig. S6b, S6d and S6f). Our results suggest that nicotine produces a blunting inhibition of serotonin neurons in freely behaving animals. Because nicotine lacked a clear effect on the Ca^2+^ signal of serotonin neurons, no correlation was observed between the change of locomotor activity with Ca^2+^ signals of serotonin neurons (Supplementary Fig. S5c and S5d).

### Cocaine suppresses the activity of dopamine neurons and serotonin neurons

**Fig. 3:**
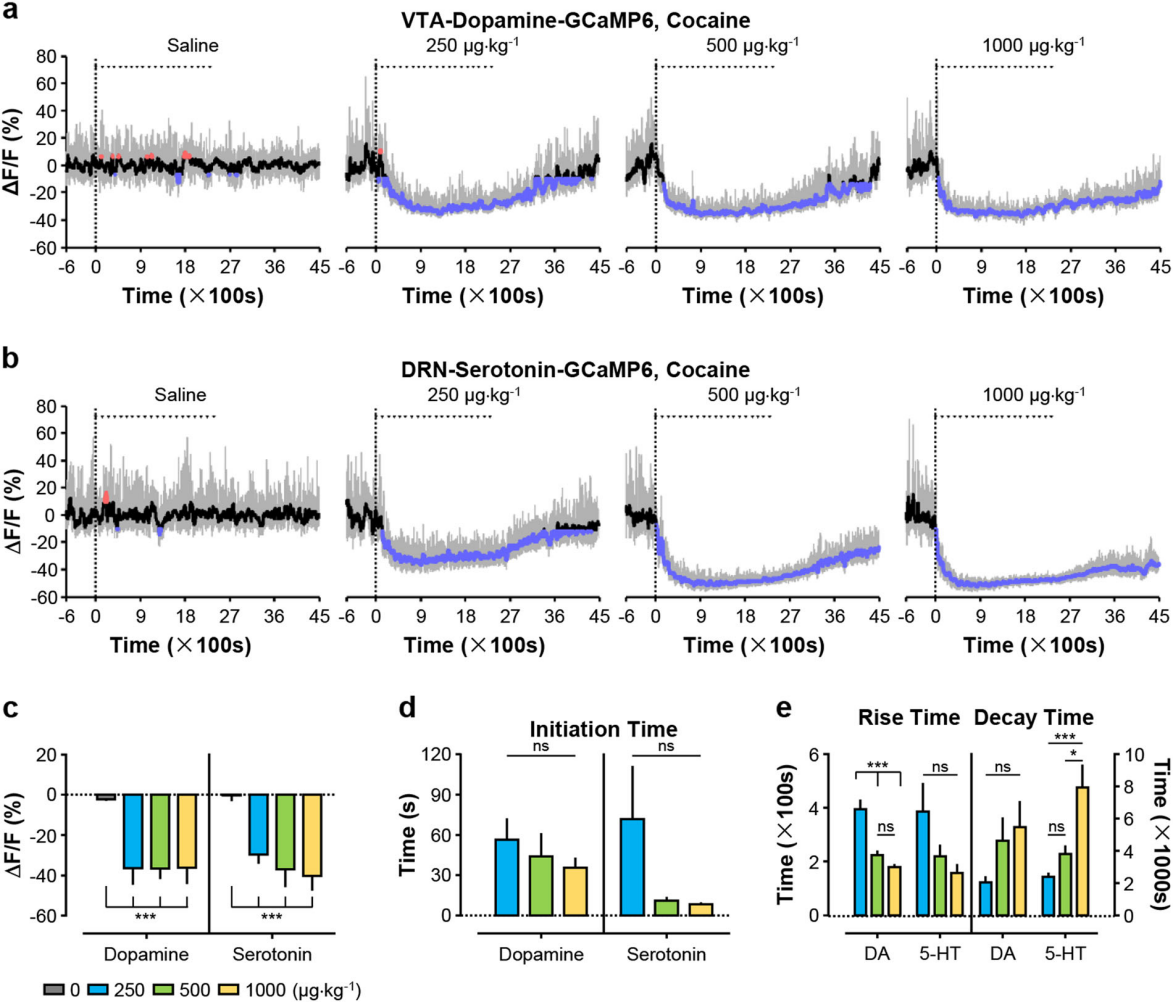
Cocaine produced strong and prolonged suppression of Ca2+ signals for both dopamine neurons and serotonin neurons. **a**, **b** Representative Ca^2+^ signals of dopamine neurons and serotonin neurons following cocaine infusion at the indicated doses. **c** Overall Ca^2+^ signals during cocaine infusion [*n* = 7 DAT-VTA-GCaMP6 mice (abbreviated as dopamine mice), F(3, 24) = 50.54, *p* < 0.0001, one-way ANOVA with Tukey’s post-hoc test; *n* = 7 Sert-DRN-GCaMP6 mice (abbreviated as serotonin mice), F(3, 24) = 56.53, *p* < 0.0001, one-way ANOVA with Tukey’s post-hoc test]. **d** Initiation time for cocaine to inhibit dopamine neurons and serotonin neurons [*n* = 7 dopamine mice, F(2, 18) = 0.5568, *p* = 0.5826, one-way ANOVA with Tukey’s post-hoc test; *n* = 7 serotonin mice, F(2, 18) = 2.446, *p* = 0.1149, one-way ANOVA with Tukey’s post-hoc test]. **e** Rise time and decay time of cocaine-induced decrease in Ca^2+^ signals of dopamine neurons and serotonin neurons [*n* = 7 dopamine mice; for rise time, F(2, 18) = 22.70, *p* < 0.0001, one-way ANOVA with Tukey’s post-hoc test; for decay time, F(2, 14) = 2.707, *p* = 0.1014, one-way ANOVA with Tukey’s post-hoc test; *n* = 7 serotonin mice; for rise time, F(2, 18) = 2.862, *p* = 0.0833, one-way ANOVA with Tukey’s post-hoc test; for decay time, F(2, 18) = 10.68, *p* = 0.0009, one-way ANOVA with Tukey’s post-hoc test]. Error bars indicate the standard error of the mean (SEM) (**c**–**e**). *p < 0.05; **p < 0.01; ***p < 0.001; ns not significant

We then examined the effect of cocaine at the doses of 0.25, 0.5, and 1.0 mg kg^−1^ per infusion corresponding to the low, median, and high doses in rodent self-administration study^31^. Cocaine at all three tested doses led to a profound dose-dependent decrease in Ca^2+^ signals (~40% decrease at the highest doses) for both VTA dopamine neurons (Fig. 3a–c) and DRN serotonin neurons (Fig. 3b, c) for over 70 min. Aligning the Ca^2+^ signals to the onset of individual drug infusions revealed that cocaine, like heroin but unlike nicotine on dopamine neurons, did not cause rapid modulation of Ca^2+^ signal of either cell-type to be precisely associated with infusion onset or offset (Supplementary Fig. S7). Cocaine also greatly reduced the amplitude and frequency of transient fluctuations of Ca^2+^ signals from VTA dopamine neurons, and completely eliminated these transient fluctuations from DRN serotonin neurons (Fig. 3a, b and Supplementary Fig. S8). Cocaine resulted in dramatic locomotor hyperactivity that was time-locked to reduction in Ca^2+^ signals from both dopamine neurons and serotonin neurons (Supplementary Fig. S9), again demonstrating that the change in neuronal activity was coupled to behavioral changes.

The decline in Ca^2+^ signals happened instantly after the first infusion began. The initiation time for cocaine-induced decrease in Ca^2+^ signals was negatively correlated with cocaine dosage, ranging from ~36 s to ~57 s for dopamine neurons (Fig. 3d) and from merely 8 s to ~72 s for serotonin neurons (Fig. 3d). Moreover, the time needed to reach the maximum decrease in Ca^2+^ signals between 150–395 s for both dopamine neurons (Fig. 3e) and serotonin neurons (Fig. 3e). Following the completion of 20 infusions, the decay of declined Ca^2+^ signals had a variable and rather long duration dependent on cocaine dosage. At the high dose of 1.0 mg kg^−1^, the decay time lasted nearly 1.5 h. for dopamine neurons (Fig. 3e) and over 2 h for serotonin neurons (Fig. 3e).

### MDMA profoundly inhibits serotonin neurons

**Fig. 4:**
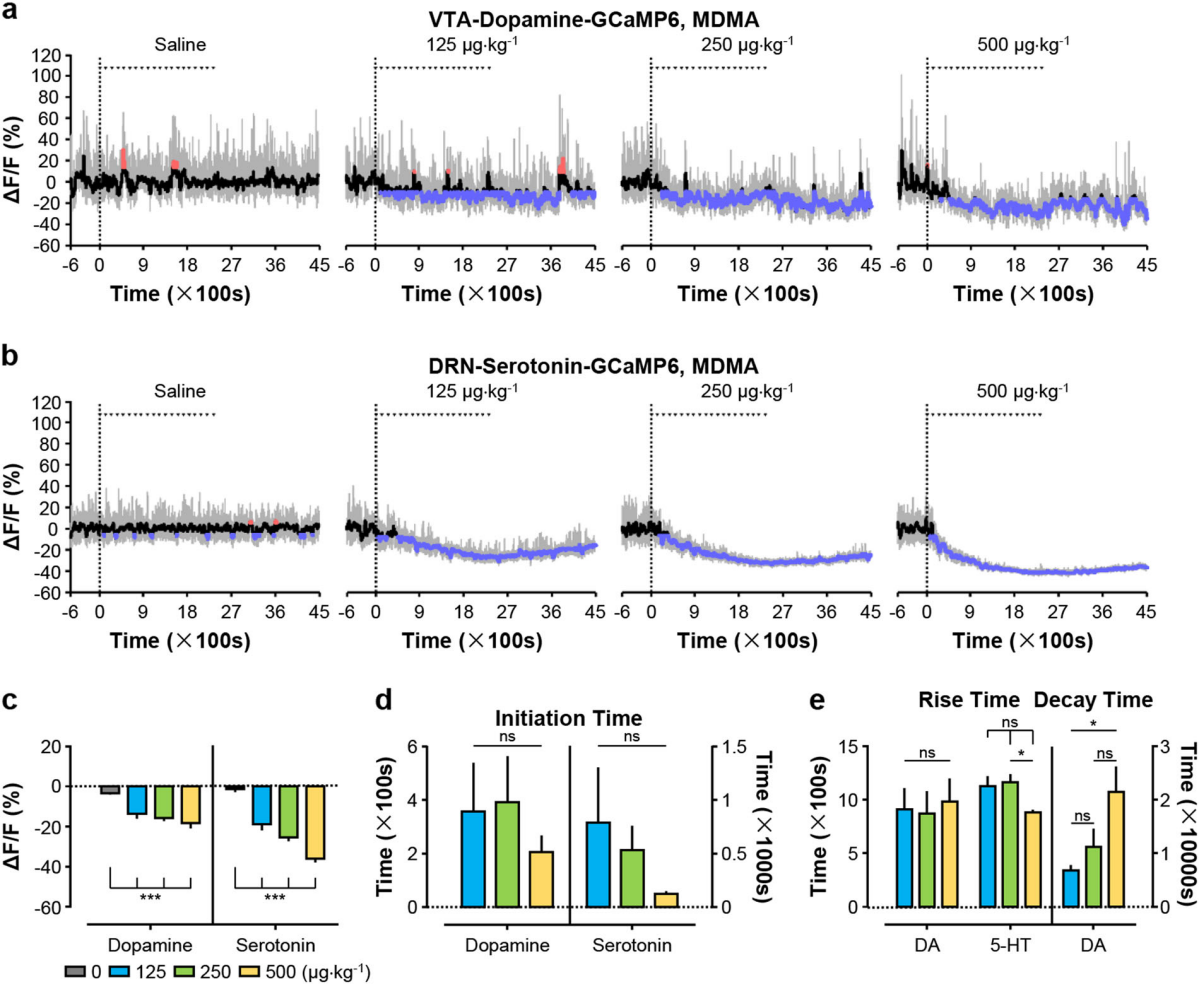
MDMA strongly reduces the activity of serotonin neurons but exerts a much weaker effect on dopamine neurons. **a**, **b** Representative Ca^2+^ signals of dopamine neurons and serotonin neurons following MDMA infusion at the indicated doses. **c** Overall Ca^2+^ signals during MDMA infusion [*n* = 6 dopamine mice, F(3, 20) = 11.58, *p* = 0.0001, one-way ANOVA with Tukey’s post-hoc test; *n* = 7 serotonin mice, F(3, 24) = 42.82, *p* < 0.0001, one-way ANOVA with Tukey’s post-hoc test]. **d** Initiation time for MDMA to inhibit dopamine neurons and serotonin neurons [*n* = 6 dopamine mice, F(2, 15) = 0.4386, *p* = 0.6529, one-way ANOVA with Tukey’s post-hoc test; *n* = 7 serotonin mice, F(2, 18) = 1.060, *p* = 0.3672, one-way ANOVA with Tukey’s post-hoc test]. **e** Rise time and decay time of MDMA-induced decrease in Ca^2+^ signals of dopamine neurons and serotonin neurons [*n* = 6 dopamine mice, F(2, 15) = 0.4386, *p* = 0.6529, one-way ANOVA with Tukey’s post-hoc test; serotonin, *n* = 7, F(2, 18) = 1.060, *p* = 0.3672, one-way ANOVA with Tukey’s post-hoc test]. Error bars indicate SEM (**c**–**e**). *p < 0.05; **p < 0.01; ***p < 0.001; ns not significant

We also tested the effects of MDMA on the activity of VTA dopamine neurons and DRN serotonin neurons in freely behaving mice. Three doses of MDMA (0.125, 0.25, and 0.5 mg kg^−1^ per infusion) were chosen according to rodent self-administration study^32^. Intravenous infusion of MDMA caused a slow and sustained decrease in Ca^2+^ signals of both dopamine neurons and serotonin neurons (Fig. 4a–c). At the highest dose, MDMA produces ~18% signal decrease in Ca^2+^ signal for dopamine neurons (Fig. 4a, c) and striking ~36% decrease for serotonin neurons (Fig. 4b, c). However, MDMA could not produce any clear effects on the short-term response of either VTA dopamine neurons or DRN serotonin neurons to repetitive infusions (Supplementary Fig. S10). Moreover, MDMA completely eliminated Ca^2+^ transients of serotonin neurons with modest effects on Ca^2+^ transients of dopamine neurons (Supplementary Fig. S11). Our results collectively show that MDMA inhibits dopamine neurons and serotonin neurons with a much stronger effect on serotonin neurons.

Unlike rapid suppression of neuron activity induced by cocaine, MDMA requires much longer time to initiate a significant decline in Ca^2+^ signals. Depending on the doses of MDMA, the initiation time was 3.5–6 min for dopamine neurons and 1–13 min for serotonin neurons (Fig. 4d). The time needed for the maximal decrease in Ca^2+^ signals was about ~15 min for dopamine neurons and ranged from ~15–19 min for serotonin neurons (Fig. 4e). Remarkably, it took several hours for Ca^2+^ signal of dopamine neurons to recover to the baseline (Fig. 4e). Unlike cocaine, MDMA did not significantly change mouse locomotor activity of DAT-Cre mice, although it had a mild but statistically significant effect on increasing the locomotor activity of SERT-Cre mice (Supplementary Fig. S12).

### Inhibitory autoreceptors mediate the responses of dopamine neurons and serotonin neurons to cocaine and MDMA

Cocaine and MDMA are thought to increase the extracellular levels of dopamine and serotonin by binding to dopamine transporter (DAT) and serotonin transporter (SERT) to block the reuptake and thus increasing the extracellular concentrations of corresponding neurotransmitters^6,33^. For dopamine neurons, extracellular dopamine then targets the inhibitory dopamine D2 receptor (DRD2) to inhibit spontaneous action potential firing, which otherwise tend to be high in freely behaving drug-naïve animals (6–10 Hz)^34–38^. However, serotonin neurons are much less active (~1 Hz)^9^. We were thus surprised by the observation that the rather silent serotonin neurons could still exhibit profound decrease in Ca^2+^ signals following cocaine and MDMA administrations. The mechanism underlying this inhibition has not been well explored. We speculated that cocaine and MDMA inhibited the activity of DRN serotonin neurons by enhancing extracellular levels of serotonin, which in turn target the inhibitory serotonin receptor 1 A (HTR1A) on serotonin neurons^39^.

**Fig. 5:**
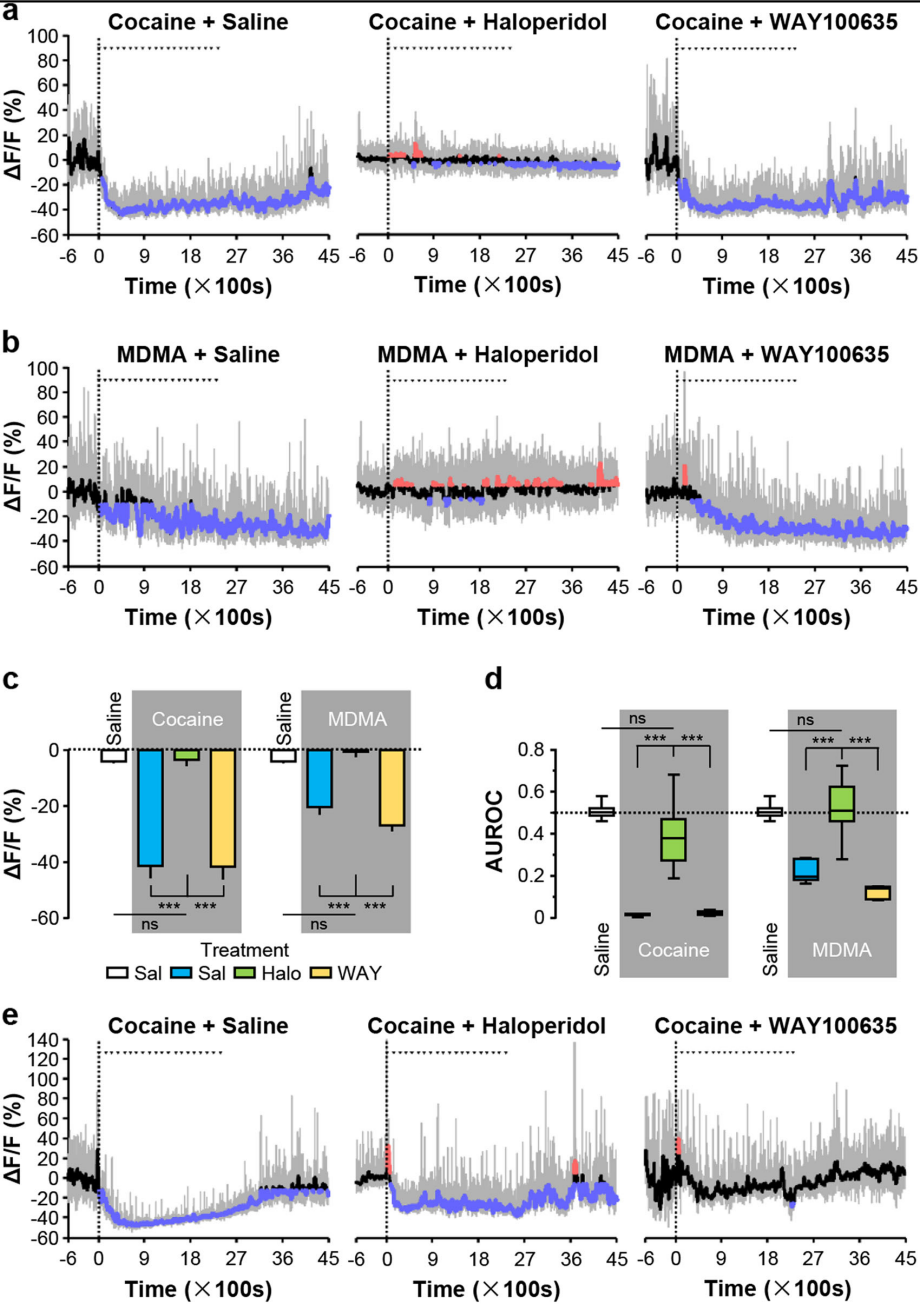

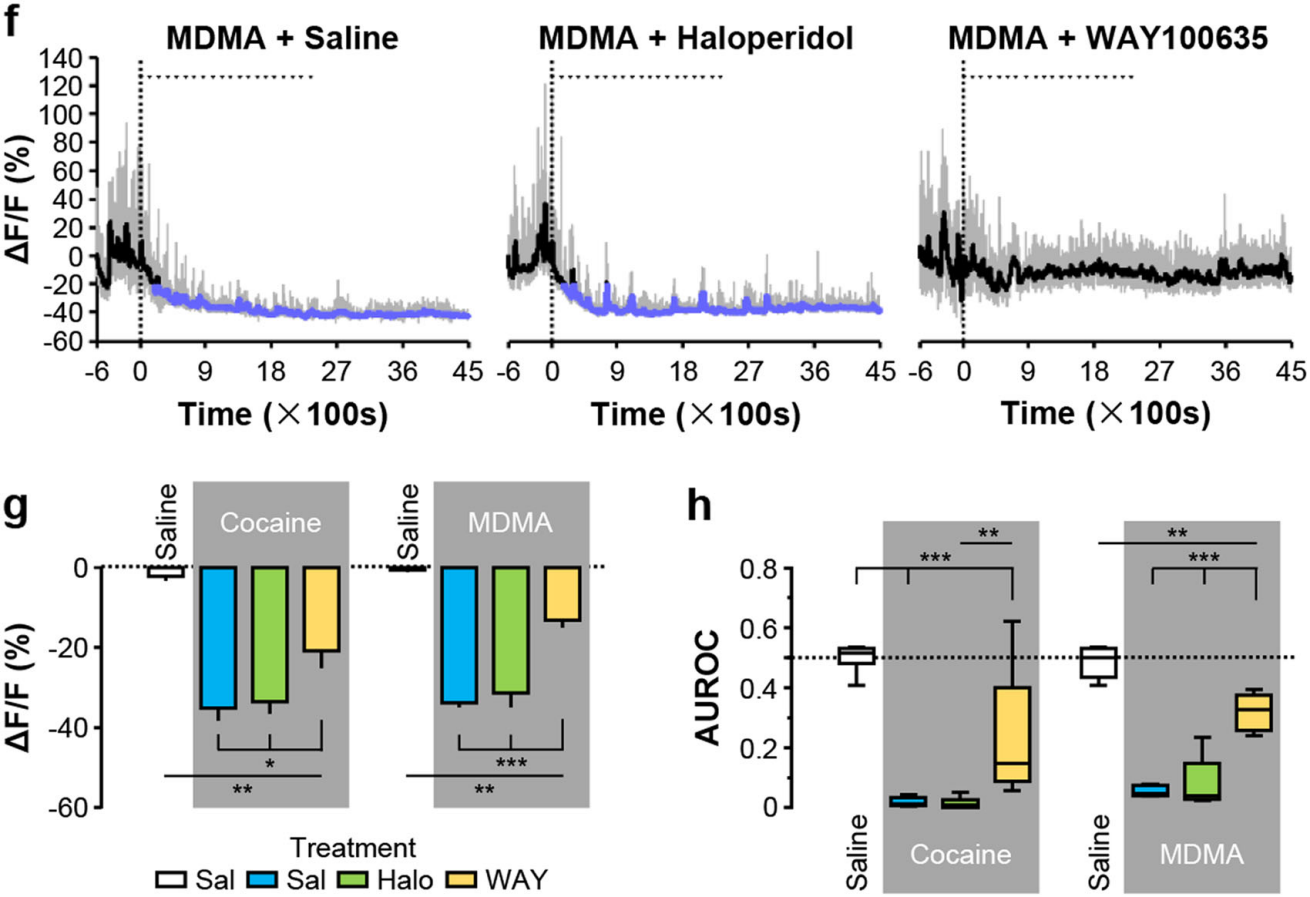
Cocaine and MDMA inhibit dopamine neurons via D2 receptor activity and inhibit serotonin neurons mainly via HTR1A receptor activity. **a**, **b** Traces showing the effects of pretreating mice with haloperidol (5 mg·kg^-1^; Halo) or WAY100635 (5 mg kg^-1^; WAY) on Ca^2+^ signals of dopamine neurons in response to cocaine (Coc) and MDMA. Saline (Sal) was infused as control. **c** The overall effect of haloperidol or WAY100635 pretreatment on the responses of dopamine neurons [*n* = 7 dopamine mice for each test group; for cocaine administration with pretreatment, F(3, 24) = 40.76, *p* < 0.0001, one-way ANOVA with Tukey’s post-hoc test; for MDMA administration with pretreatment, F(3, 24) = 39.24, *p* < 0.0001, one-way ANOVA with Tukey’s post-hoc test]. **d** ROC analysis measuring the effect of haloperidol and WAY100635 on the inhibitory responses to cocaine or MDMA [the same group size as in (**c**) for cocaine administration with pretreatment, F(3, 24) = 65.44, *p* < 0.0001, one-way ANOVA with Tukey’s post-hoc test; for MDMA administration with pretreatment, F(3, 24) = 47.54, *p* < 0.0001, one-way ANOVA with Tukey’s post-hoc test]. **e**, **f** Representative traces of from serotonin neurons showing the effect of pretreating mice with haloperidol or WAY100635 on the responses to cocaine or MDMA. **g** The overall effect of haloperidol or WAY100635 pretreatment on the responses of serotonin neurons [*n* = 9 serotonin mice for Sal-Sal and Sal + Cocaine, *n* = 6 for Halo + Coc, *n* = 8 for WAY + Coc, F(3, 26) = 24.02, *p* < 0.0001, one-way ANOVA with Tukey’s post-hoc test; *n* = 5 for Sal + Sal, Sal-MDMA, Halo + MDMA, and WAY + MDMA, F(3, 16) = 55.60, *p* < 0.0001, one-way ANOVA with Tukey’s post-hoc test]. **h** ROC analysis to discriminate among the effects of haloperidol and WAY100635 on cocaine-induced and MDMA-induced inhibition [the same group size as in (**c**) for cocaine administration with pretreatment, F(3, 28) = 39.52, *p* < 0.0001, one-way ANOVA with Tukey’s post-hoc test; for MDMA administration with pretreatment, F(3, 16) = 57.62, *p* < 0.0001, one-way ANOVA with Tukey’s post-hoc test]. Error bars indicate SEM (**c**, **d**, **g**, **h**). *p < 0.05; **p < 0.01; ***p < 0.001; ns not significant

We first confirmed that the DRD2 antagonist haloperidol could block the inhibitory effects of cocaine and MDMA on the activity of dopamine neurons. Pretreating mice with an intraperitoneal injection of haloperidol completely prevented the inhibitory effects of cocaine and MDMA on the activity of dopamine neurons, whereas no such preventative effect was observed when animals were pretreated with saline (control) or WAY100635 (an inhibitor of HTR1A) (Fig. 5a–d and Supplementary Fig. S13). Together, we demonstrated that cocaine- and MDMA-induced inhibition of dopamine neurons was mediated through DRD2.

We next tested whether HTR1A was responsible for the inhibition of serotonin neurons caused by cocaine and MDMA. Consistent with our hypothesis, cocaine- and MDMA-induced inhibition of serotonin neurons were substantially blocked by pretreatment with WAY100635, but not by the pretreatment with saline or haloperidol (Fig. 5e–h and Supplementary Fig. S14). Collectively, these results substantiated the role of HTR1A in mediating the inhibitory response of serotonin neurons to cocaine and MDMA.

## Discussion

Heroin, nicotine, cocaine, and MDMA are commonly abused drugs that are believed to target neurons involved in reward processing. Considering that rapid change in the activity of dopamine and serotonin neurons could have profound effect on animal behaviors and the state of anesthesia fundamentally influences the activity of these neurons^40–42^, it is important to study how drugs of abuse affect the activity of these two neuron populations in freely behaving animals. Using fiber photometry of Ca^2+^ signals, we demonstrated that these drugs potently and differentially modulate the activity of VTA dopamine neurons and DRN serotonin neurons in freely behaving mice. Our results have several functional implications on how drugs may produce different behavioral effects by distinctly affecting the activity of dopamine neurons and serotonin neurons. Moreover, the method of combining fiber photometry and intravenous drug delivery may well complement current approaches, including microdialysis, FSCV and electrophysiology, for unraveling rapid impacts of the wide-ranging drugs.

We found that heroin strongly activates VTA dopamine neurons in behaving mice. We also provide the important information regarding the dose-kinetic relationship. Intensive research has hypothesized that opiate activates µ-opioid receptors on local and VTA-projecting GABA neurons and then disinhibits dopamine neurons^22,24^. However, the finding of sustained activation of dopamine neurons in vivo has not been observed by classic electrophysiology^22,24^. It is an interesting question whether this long-term activation of dopamine neurons is correlated with self-administration performance. Very few studies have studied the in vivo responses of DRN serotonin neurons to opiate exposure. We show that heroin at higher doses also significantly activates serotonin neurons. This heroin-induced activation may be achieved by disinhibiting serotonin neurons through reducing local GABAergic activity^43,44^. Given that some DRN serotonin neurons may corelease glutamate^8,45^, some heroin-associate behaviors might be mediated by serotonin and the potentially coreleased glutamate.

Nicotine potently activates VTA dopamine neurons but lacks a strong effect on DRN serotonin neurons. The excitatory response of dopamine neurons to nicotine is consistent with earlier electrophysiological recordings from brain slice preparations^21,46,47^ and FSCV from behaving animals^18,48^. Here we provide the first evidence that nicotine requires only 2.2–4.4 s to initiate the responses and 16–19 s to reach the peak activation level. This speed is impressive considering that nicotine needs to cross blood–brain barrier and diffuse to the VTA before activating dopamine neurons. Rapid activation of dopamine neurons may facilitate reinforcement of tobacco smoking. We showed that in freely behaving animals the initial infusion of nicotine activates dopamine neurons while later repetitive infusions produce an inhibition and then rebound response pattern. This inhibition-rebound pattern is consistent with previous recordings from anesthetized animals^49,50^. The exact mechanisms underlying this biphasic pattern remain unclear: it is possible that nicotine may transiently activate GABA neurons that in turn inhibit dopamine neurons^51^. The inhibition of dopamine neurons produces behavioral aversion^52,53^, suggesting that the transient inhibitory effect of nicotine may be associated with its aversive quality in some people. Unlike VTA dopamine neurons, DRN serotonin neurons do not increase Ca^2+^ signals following intravenous infusions of nicotine at the doses of up to 0.2 mg kg^−1^. Moreover, nicotine blocks Ca^2+^ transients of serotonin neurons, suggesting a possibly inhibitory effect. This observation differs from an early microdialysis study showing that acute administration of nicotine at high doses (up to 8 mg kg^−1^) enhances the extracellular level of serotonin^54^. The apparent discrepancy between the present study and early reports might be reconciled by dose difference, the potential stimulant effect of nicotine on presynaptic terminals but not somata of serotonin neurons, and/or technical difference between fiber photometry and microdialysis.

This study provides the first demonstration that cocaine causes strong and sustained inhibition of both dopamine neurons and serotonin neurons in behaving state. This result substantiates early observations using single-unit recording in anesthetic animals that psychostimulants decrease the firing rate of dopamine neurons^35,38^. We further revealed that both dopamine neurons and serotonin neurons undergo the long-lasting inhibition from MDMA exposure, with serotonin neurons exhibiting a much greater extent of inhibition. This is consistent with the concept that cocaine and MDMA enhances extracellular levels of dopamine and serotonin by binding to DAT and SERT, thus blocking their reuptake. MDMA binds more weakly to DAT than to SERT^33^, which explains a milder effect of MDMA on dopamine neurons. The increased levels of dopamine and serotonin then in turn acts on inhibitory autoreceptors to inhibit dopamine neurons and serotonin neurons through the opening of GIRK-type potassium channels^34,55^.

Our findings raise an intriguing question: how can cocaine and MDMA cause massive release of dopamine and serotonin in light of the prolonged (~20 min to ~6 h) suppression of neuronal activity? We propose the following four possible mechanisms: (1) Dopamine and serotonin can be released via an action-potential-independent pathway upon drug exposure. Indeed, cholinergic inputs can bypass activity in dopamine neurons and drive dopamine release^56^. Similarly, serotonin release can be evoked by the activation of glutamate receptors and opening of l-type Ca^2+^ channel independent of action potential^57^. (2) Upon drug exposure, a majority of neurons experience strong inhibition while a minority of neurons are actually activated to maintain the high level of extracellular dopamine and serotonin. The neuronal response of the latter ones cannot be detected by the fiber photometry at the population level. Indeed, a recent study reported that a subpopulation of VTA dopamine neurons were activated by acute cocaine exposure^58^. (3) The auto-inhibition is secondary to the blockade of dopamine and serotonin reuptake. (4) Without the reuptake by transporters, extracellular dopamine and serotonin cannot be efficiently metabolized and remain effective for a long period.

The strong inhibitory effects of cocaine and MDMA on the activity of dopamine neurons and serotonin neurons have several functional implications. First, previous studies focus on the effect of cocaine on dopamine levels^59^ and that of MDMA on serotonin levels^60^. Our data indicate that we should also consider the role of serotonin in cocaine-induced behaviors and that of dopamine in MDMA-induced behaviors. At least, some animal studies suggest a role of serotonin transporters in cocaine-related reward behaviors^11–13^. Second, VTA dopamine neurons and DRN serotonin neurons respond phasically to rewards and reward predicting cues^9,10^. It is interesting to test whether cocaine or MDMA would prevent these neurons from rapidly responding to external reward-related signals. Thus far this issue has not been fully resolved. Acute exposure of cocaine would enhance the dopamine release within NAc shell during a cue-cocaine Pavlovian training^61^. However, chronic use of cocaine would dramatically dampen the outflow of dopamine within NAc responding to drug-delivered cues, which then promotes the escalation of cocaine intake^62^. Third, glutamate is coreleased from subsets of dopamine neurons^63,64^ and serotonin neurons^8,45^. Cocaine could thus enhance extracellular dopamine and serotonin, and simultaneously suppress glutamate corelease owing to the auto-inhibition, which may underpin certain drug-associated behaviors.

We note a possible association between drug-induced reinforcing property and the drug effects on dopamine neurons and that between drug-induced euphoria property and the drug effects on serotonin neurons. Heroin, nicotine, and cocaine produce powerful dependence in humans^2^, we show that they either directly activate dopamine neurons or inhibit dopamine neurons likely by causing massive release of dopamine. By contrast, MDMA produces much weaker drug dependence than cocaine^2^, we also show that it has weaker effect than cocaine on dopamine neurons. These data are consistent with the “Dopamine Hypothesis” of drug addiction. Moreover, heroin^65^, cocaine^66^, and MDMA^67^ users often report a surge of euphoria; we show that they strongly excite serotonin neurons or promote serotonin release. By contrast, nicotine only produces mild euphoria for some people^68^; we show that it has no significant effect on serotonin neurons. These observations suggest that massive release of serotonin but not dopamine may be more relevant to the euphorigenic properties of many drugs of abuse.

Current views of drug abuse propose a unitary theory of addiction based on the midbrain dopamine system. However, more balanced researches are in need with careful characterization of similarities and differences in neurophysiological changes induced by different classes of drugs^69^. Here we revealed that different drugs dramatically and differentially regulate the activity of both VTA dopamine neurons and DRN serotonin neurons, suggesting that drugs of abuse could have distinct behavioral impacts in addition to their common effects on dopamine release. Especially, the opposing effects of heroin and cocaine over the activity of VTA dopamine neurons may help understand the opposite effects on the morphology of dopamine neurons^70^ and the differential engagement of direct and indirect pathways^69^ across opiates and psychostimulants. In future studies, monitoring the response of VTA dopamine neurons and DRN serotonin neurons within the context of drug self-administration may reveal similarities and differences across different drugs to interact with internal state and drug-taking environment to induce addictive-like behaviors^71^.

## Materials and Methods

Experimental procedures were approved by the National Institute of Biological Sciences (NIBS), Beijing in accordance of the regulations of the Administration of Affairs Concerning Experimental Animals of China.

### Animals

*DAT-Cre* mice (strain name B6.SJL-Slc6a3tm1.1(cre) Bkmn/J, Jackson Laboratory, Bar Harbor, Maine, USA) and *Sert-Cre* mice (strain name B6.Cg-Tg(Slc6a4-Cre)ET33Gsat, Mutant Mouse Resource and Research Center, USA) were backcrossed onto a C57BL/6 N background (Vitalriver Laboratory Animals, Beijing, China) in NIBS animal facilities. All mice were housed in cages of 1–5 on a 12/12 light/dark cycle, and were at least at age of 8 weeks when any experiment began. Mice were maintained in an environmentally controlled vivarium and given ad libitum access to food and water. Before the beginning of experiment, mice had been kept on a reverse light/dark cycle (light off at 8:00 am) for at least 1 week.

### Drugs

For intravenous infusion, all drugs were dissolved in 0.9% sterile saline including (-)-nicotine hydrogen tartrate salt (nicotine; Sigma-Aldrich, China), heroin, cocaine hydrochloride (cocaine) and 3,4-Methylenedioxymethamphetamine (MDMA) hydrochloride. The pH of solutions for intravenous infusion was adjusted to ~7.4. The doses per infusion for each drugs were adjusted according to animal weight: (1) 0.025, 0.05, and 0.1 mg kg^−1^ for heroin; (2) 0.06, 0.1, and 0.2 mg kg^−1^ for nicotine; (3) 0.25, 0.5, and 1.0 mg kg^−1^ for cocaine; (4) 0.125, 0.25, and 0.5 mg kg^−1^ MDMA. Note that the doses of heroin and nicotine referred to the free-base form while the rest referred to the salt form. For intraperitoneal injection, WAY100635 (WAY; Sigma-Aldrich, USA) was dissolved in 0.9% saline. Haloperidol (Halo; Sigma-Aldrich, China) was first dissolved in DMSO and then diluted with 0.9% saline. Each mouse was intraperitoneally pretreated with either 5 mg kg^−1^ haloperidol to block DRD2 or 5 mg kg^−1^ WAY100635 to block HTR1A.

### AVV vectors

AAV serotype2/9 carrying DIO-GCaMP6m plasmid or DIO-EmGFP plasmid was produced in house with the final titers up to 1–5 × 10^12^ particles per ml. GCaMP6m (Plasmid 40754, Addgene, Massachusetts, USA) or enhanced membrane GFP (Plasmid 14757, Addgene) was cloned into the pAAV-EF1 a-DIO-hChR2(H134R)-mCherry construct (a gift from K. Deisseroth) by replacing the segment encoding ChR2-mCherry.

### Surgeries

Each mouse received the following two surgeries: (1) Virus injection & optical fiber implantation; and (2) Intravenous catheterization surgery.

### Virus injection and optical fiber implantation

Virus injection and fiber implantation was performed as described previously^8–10,20^. Mice were anaesthetized with an intraperitoneal injection of 250 mg·kg^−1^ avertin (2,2,2-tribromoethanol, Sigma-Aldrich, UK) and mounted onto a stereotaxic instrument (RWD Life Science, Shenzhen, China) on a heat pad with ophthalmic ointment applied to each eyes. The scalp was shaved and then incised through the midline. A craniotomy was made by gently drilling a small hole on the skull. By a microsyringe pump (Nanoliter 2000 Injector, WPI, Florida, USA), AAV vectors (500 nL) was slowly infused through a sharp glass pipette into the target brain area at rate of 46 nL·min^−1^. The glass pipette remained still at the injection site for another 5 min after the infusion was completed, and then were slowly withdrawn. For *DAT-Cre* mice, virus was infused in the ventral tegmental area [3.2 mm anteroposterior (AP), 0.5 mm mediolateral (ML), 4.2 mm dorsoventral (DV) relative to the bregma]. For *Sert-Cre* mice, virus was infused in the dorsal raphe (5.0 mm AP, 0 mm ML, 2.5 mm DV tilted at an angle of 15° toward the caudal).

Immediately following virus infusion, an optical fiber [200 μm outer diameter (O.D.), 0.39 numerical aperture (NA), Thorlabs, Massachusetts, USA] bounded to a ceramic ferrule was implanted with its tip targeting at the virus injection site. Ceramic ferrule were secured to the skull using dental acrylic with the help of a skull-penetrating M1 screw. Mice were gently removed from the stereotaxic instrument and placed over a heat pad in their home cages. With daily monitoring for wound healing and body weight, mice were singly housed and allowed to recover for at least 1 week.

### Intravenous catheterization surgery

After 1 week of recovery, mice were tested for fluorescence intensity. Only those showing fluctuation in fluorescence intensity at least up to 10% (*ΔF*/*F*) during environmental exploration were collected for catheterization of jugular vein. During chronic fiber photometry, Ca^2+^ signals in some cases gradually decreased across days possibly because of phototoxicity. We excluded the data from mice exhibiting exploration-associated Ca^2+^ signal fluctuations below 10% (*ΔF*/*F*). Catheters consisted of three parts, including a 60-mm length of renathane tubing [0.03 mm inner diameter (I.D.), 0.06 mm O.D., Braintree Scientific, Massachusetts, USA], a steel cannula (0.03 mm I.D., 0.05 mm O.D., Anilab, Zhejiang, China) and a 30-mm length of polyethylene tubing (0.04 mm I.D., 1.1 mm O.D., Anilab). The steel cannula was bent at a right angle with one end inserted into the renathane tubing and the other one into the polyethylene tubing.

Cannulation surgery were performed as described previously with adaptive modifications^30,72^. Mice were anaesthetized with of 250 mg kg^−1^ avertin. A 10-mm length of the renathane tubing was inserted into the right external jugular vein of mouse and secured with surgical silk suture around the silicone knot (SILASTIC^®^ Medical Adhesive Silicone Type A, Dow Corning, Michigan, USA) located 10 mm away from the tubing tip. The rest of renathane tubing was subcutaneously passed through the animal’s neck to the skull. The bent steel cannula was secured to the skull using dental acrylic enough away from the previously implanted ferrule. The free end of polyethylene tubing was blocked by blunt steel nail. Catheters were daily flushed with heparin saline solution (30 units per ml; J&K, Beijing, China). Catheter patency was verified with the short-acting anesthetic etomidate (2 mg ml^−1^; J&K, Beijing, China) when necessary and at the end of all experiments. We excluded data from mice that failed in catheter patency test from further data analysis. With daily monitoring for wound healing and body weight, mice were singly housed and allowed with at least 48 h to recover for further experiments.

### Fiber photometry

A setup for fiber photometry of GCaMP signals was constructed as described previously^9,10,73^ (Fig. 1a). Briefly, an Optically-Pumped Semiconductor laser (OBIS 488LS, Coherent, California, USA) producing laser beam of 488-nm wavelength was used as the excitation source. The laser beam was reflected by a dichroic mirror (MD498, Thorlabs) and coupled into an optical commutator (FRJ_1 × 1_FC-FC, Doric Lenses, Quebec, Canada) through a 10× objective lens (0.3 NA; Olympus, Japan). A 2-m length of optical fiber (200 μm O.D., 0.39 NA, Thorlabs) was used to transmit light between the optical commutator and the implanted optical fiber. Note that we used a single optical commutator to prevent rotation-induced noises, and used 1 m optical fiber and 1 m flexible tubing for drug delivery to facilitate mouse movement. The laser intensity was measured at the tip of optical fiber and adjusted to ~0.02 mW to alleviate photobleaching. The laser beam delivered by the optical fiber to excite GCaMP6m expressed in the target brain area. GCaMP6m fluorescence was collected by the optical fiber, passed through the dichroic mirror, filtered through a bandpass filter (MF525–39, Thorlabs), and projected onto a photomultiplier tube (R3896, Hamamatsu Photonics, Shizuka, Japan) where light intensity was converted into current signal. An amplifier (C7319, Hamamatsu Photonics) was used to convert the current signal to voltage signal, which was further directed to a low-pass filter (40-Hz cut-off; Model 440, Brownlee Precision, California, USA) to allow filtering of noise at higher frequency. Finally, the analogue voltage signal was digitalized at 500 Hz and recorded by a Power 1401 digitizer and Spike2 software (CED, Cambridge, UK).

### Intravenous drug delivery

A mouse was placed in a chamber (200 × 200 × 220, Length × Width × Height in mm), and allowed to passively receive drugs and simultaneously recorded GCaMP6m fluorescence (Fig. 1a). A peristaltic pump (Anilab) delivered drug solution through the tubing into the right jugular vein. The pump was controlled through an IC board (Arduino Uno R3) using a self-developed MATLAB program (MathWorks, Massachusetts, USA). Every time the pump was triggered, the program would generate a timing signal to a Power 1401 digitizer in order to synchronize with recording of GCaMP6m fluorescence change.

We tested mice with multiple drugs in the following order: saline, cocaine, heroin, MDMA, and nicotine. On day 1, mice were challenged with saline as control. We allow mice to rest 4 days after each drug to wean off potential effects of prior drug history on the response to the subsequent drug. For each drug, mice were randomly assigned into two groups, with one group treated with the increasing doses and the other with the decreasing doses over consecutive days. On each day, a drug delivery session consisted of four phases, including a 10 min habituation phase, a 10 min pre-infusion phase, a 40 min infusion phase, and a 35 min post-infusion phase. During the habituation phase, mice was allowed 10 min to habituate the environment and GCaMP6m signals became largely stabilized. In the pre-infusion phase, GCaMP6m fluorescence was recorded as the control. During the infusion phase, 20 infusions of a drug (speed 7 μL s^−1^, duration 2 s) were performed every 2 min. Finally in the post-infusion phase, the infusion was completed and GCaMP6m signals continued to be recorded.

### Immunohistochemistry

Mice were anaesthetized with 250 mg kg^−1^ avertin and transcardially perfused with 0.9% saline, followed by 4% paraformaldehyde dissolved in 0.1 M phosphate buffer saline (PBS). Brains were harvested, fixed for 4 h in 4% paraformaldehyde, and immersed in 30% sucrose for 2 days. The brains were cut into 30-μm coronal sections on a cryostat (Leica CM1900, Germany). Floating sections were washed for 5 min 3 times in PBS with 0.3% Triton X-100 (PBST), and then blocked by 3% Bovine Serum Albumin in PBST. Subsequently, sections were incubated with primary antibody in PBST overnight at 4 °C. After washing in PBST, sections were incubated with secondary antibody in PBS for 1 h at room temperature. After washing again, sections were cover-slipped 50% glycerol mounting medium. Image data were acquired and digitalized using a confocal microscope (Zeiss LSM510 Meta, Germany). Primary antibodies used were: rabbit-anti-GFP (1:500, thermos fisher scientific), chicken-anti-GFP (1:500, Abcam), rabbit-anti-TH (1:500, Merk Millipore), and rabbit-anti-TPH2 (1:500, Merk Millipore). Secondary antibodies used were: fluorescein-conjugated goat-anti-rabbit (1:500, Jackson ImmunoReseach), fluorescein-conjugated donkey-anti-chicken (1:500, Jackson ImmunoResearch), and Cy3-conjugated goat-anti-rabbit (1:500, Jackson ImmunoResearch).

### Fiber photometry analysis and statistical tests

Analysis of fiber photometry data was conducted with custom-written MATLAB programs. Statistical tests for one-way was performed with Graph Pad Prism (version 6, California, USA).

### ΔF/F calculation

Raw data of fiber photometry were converted into MATLAB Mat files using Spike2 for further processing. The change in GCaMP6m fluorescence intensity (*ΔF/F*) was calculated by the function *ΔF/F* = (*F*  – *F*_0_) / *F*_0_, where *F*_0_ was derived from the mean value of data points: (1) within an entire pre-infusion phase for analysis of long-term response to drug exposure; (2) within a 1.5 s window flanking the −0.5 s time point before each infusion for analysis of short-term response to drug exposure. *ΔF/F* values were presented in average plots.

### Extraction of the baseline from long-term calcium signals

The baseline was obtained by smoothing raw GCaMP6m fluorescence with the MATLAB *medfilt1* function (30 s span, one-dimensional median filter) to minimize transient changes.

### Correction for photobleaching

Due to the nature of photobleaching associated with long-term recording, GCaMP6 fluorescence intensity displayed a gradually decreasing trend. Photobleaching correction for intra-infusion response was not necessary because of the short time window (62 s) during which photobleaching, if any, was negligible. To measure and correct for photobleaching during the entire recording session (85 min), we used a two-step protocol (Supplementary Fig. S1d). The nature of our protocol was based on an assumption that saline treatment had no significant effects over neuronal activities and GCaMP6m fluorescence. Therefore, as to saline administration, the decreasing magnitude of fluorescence intensity could represent photobleaching.

Step 1: A baseline was extracted from the original GCaMP6m fluorescence exposed to saline as described previously. We then applied the MATLAB *polyfit* function (6^th^ degree, polynomial fitting) to the baseline to obtain the smooth photobleaching curve.

Step 2: To obtain the photobleaching-corrected GCaMP6m fluorescence, the photobleaching curve were subtracted from original fluorescence signals that collected from either saline or drug infusion.

### Permutation test for the baseline of long-term GCaMP6 signals

A simple permutation test was used to determine whether significant changes occurred along a baseline of GCaMP6 fluorescence upon drug exposure. To achieve this purpose, a control distribution of data points from the fluorescence baseline during the pre-infusion phase was obtained. Then, *α*-level was set as 0.05 to compare each data point of the fluorescence baseline during the infusion phase and the post-infusion phase with the control distribution. Statistical significance at each time point was generated when data points were larger than the 95th percentile or smaller than 5th percentile of the control distribution. Finally, red or blue color was superimposed over the fluorescence baseline to highlight significant increase or decrease, respectively.

### Calculation of initiation time

Initiation time was the time when drugs were starting to significantly increase or decrease the baseline of GCaMP6 fluorescence. Specifically, we took the first occurrence of three consecutive time points, at each of which GCaMP fluorescence levels were consistently significantly increased or decreased, as the initiation time point.

### Calculation of rise time and decay time

A rise phase consisted of two parts, a pre-infusion phase and an infusion phase. A baseline of GCaMP6 fluorescence within the rise phase was modeled by 20^th^-degree polynomial fitting (MATLAB *polyfit* function) to yield a rise curve. An amplitude of the rise curve could be calculated by subtracting a low-state level (the *ΔF/F* value at the start point of the rise curve) from a high-state level (the maximum value of increasing rise curve or the minimum value of decreasing rise curve). Reference levels were a percentage of the amplitude, including 20 and 80% reference levels. Rise time was the time required for the rise curve to cross from the 20% up to 80% reference levels.

A decay phase was composed of the last 10 min of the infusion phase and a post-infusion phase. Unlike rise curve, a decay curve was obtained by using an exponential model (MATLAB *fit* function) to fit the fluorescence baseline within the decay phase. Either one-term or two-term exponential models were chosen according to fitting property. Decay time was defined as the time taken by the decay curve to change from the 80% back to 20% reference levels.

### Peak Analysis

Generally, raw GCaMP6m fluorescence was forced to detrend in order to reduce the overall variation, and then the fluorescent transients was extracted and analyzed. Specifically, raw GCaMP6m fluorescence were detrended with MATLAB *msbackadj* function. In order to determine the frequency and amplitude of fluorescent transients, the MATLAB *findpeaks* function with the ‘MinPeakProminence’ parameter set as 5, was applied to detect the local peaks (*ΔF/F*) of the detrended GCaMP6m fluorescence. Only a local maximum prominently stands out at least 5% higher than the nearby fluorescence intensity were taken as the peak of a fluorescent transient. The frequency of fluorescent transients were defined as the number of detected peaks within 10 min. The amplitude of fluorescent transients were presented with cumulative graphs.

### ROC Analysis

We performed the receiver operating characteristic (ROC) analysis to verify the strength of activation or inhibition. The distributions of GCaMP6m fluorescence intensity within a pre-infusion phase or an infusion phase was obtained in the first place. A ROC curve was drawn, relating the proportion of hits (true positive ratio) to false alarm (false positive ratio) across a range of confidence levels. Finally, the strength of a ROC curve was generated by calculating the area under the ROC curve (AUROC), the value of which is equal to 0.5, indicating no response, larger than 0.5 indicating activation and smaller than 0.5 indicating inhibition. AUROCs were presented with bar graphs.

## Electronic supplementary material

Supplementary Information

## Electronic supplementary material

**Supplementary Information** accompanies the paper at (10.1038/s41421-018-0060-z).

## References

[1] United Nations Office on Drugs and Crime. *World Drug Report 2016* (United Nations publication, NY, 2016).

[2] Nutt D, King LA, Saulsbury W, Blakemore C (2007). Development of a rational scale to assess the harm of drugs of potential misuse. Lancet.

[3] Parrott AC, Lasky J (1998). Ecstasy (MDMA) effects upon mood and cognition: before, during and after a Saturday night dance. Psychopharmacology.

[4] Hung RJ (2008). A susceptibility locus for lung cancer maps to nicotinic acetylcholine receptor subunit genes on 15q25. Nature.

[5] Nestler EJ (2005). Is there a common molecular pathway for addiction?. Nat. Neurosci..

[6] Gether U, Andersen PH, Larsson OM, Schousboe A (2006). Neurotransmitter transporters: molecular function of important drug targets. Trends Pharmacol. Sci..

[7] Zhu Y, Wienecke CF, Nachtrab G, Chen X (2016). A thalamic input to the nucleus accumbens mediates opiate dependence. Nature.

[8] Liu Z (2014). Dorsal raphe neurons signal reward through 5-HT and glutamate. Neuron.

[9] Li Y (2016). Serotonin neurons in the dorsal raphe nucleus encode reward signals. Nat. Commun..

[10] Zhong W, Li Y, Feng Q, Luo M (2017). Learning and stress shape the reward response patterns of serotonin neurons. J. Neurosci..

[11] Land BB (2009). Activation of the kappa opioid receptor in the dorsal raphe nucleus mediates the aversive effects of stress and reinstates drug seeking. Proc. Natl Acad. Sci. USA.

[12] Bruchas MR (2011). Selective p38alpha MAPK deletion in serotonergic neurons produces stress resilience in models of depression and addiction. Neuron.

[13] Rocha BA (1998). Increased vulnerability to cocaine in mice lacking the serotonin-1B receptor. Nature.

[14] Harris GC, Aston-Jones G (2001). Augmented accumbal serotonin levels decrease the preference for a morphine associated environment during withdrawal. Neuropsychopharmacology.

[15] Tanda G, Pontieri FE, Di Chiara G (1997). Cannabinoid and heroin activation of mesolimbic dopamine transmission by a common mu1 opioid receptor mechanism. Science.

[16] Doly S (2008). Serotonin 5-HT2B receptors are required for 3,4-methylenedioxymethamphetamine-induced hyperlocomotion and 5-HT release in vivo and in vitro. J. Neurosci..

[17] Phillips PE, Stuber GD, Heien ML, Wightman RM, Carelli RM (2003). Subsecond dopamine release promotes cocaine seeking. Nature.

[18] Cheer JF (2007). Phasic dopamine release evoked by abused substances requires cannabinoid receptor activation. J. Neurosci..

[19] Vander Weele CM (2014). Rapid dopamine transmission within the nucleus accumbens: dramatic difference between morphine and oxycodone delivery. Eur. J. Neurosci..

[20] Zhang J (2016). Presynaptic excitation via GABAB receptors in habenula cholinergic neurons regulates fear memory expression. Cell.

[21] Picciotto MR (1998). Acetylcholine receptors containing the beta2 subunit are involved in the reinforcing properties of nicotine. Nature.

[22] Johnson SW, North RA (1992). Opioids excite dopamine neurons by hyperpolarization of local interneurons. J. Neurosci..

[23] Bocklisch C (2013). Cocaine disinhibits dopamine neurons by potentiation of GABA transmission in the ventral tegmental area. Science.

[24] Jalabert M (2011). Neuronal circuits underlying acute morphine action on dopamine neurons. Proc. Natl Acad. Sci. USA.

[25] Chen TW (2013). Ultrasensitive fluorescent proteins for imaging neuronal activity. Nature.

[26] Gunaydin LA (2014). Natural neural projection dynamics underlying social behavior. Cell.

[27] Cui G (2013). Concurrent activation of striatal direct and indirect pathways during action initiation. Nature.

[28] Wang S, Tan Y, Zhang JE, Luo M (2013). Pharmacogenetic activation of midbrain dopaminergic neurons induces hyperactivity. Neurosci. Bull..

[29] Wang XF (2016). T394A mutation at the mu opioid receptor blocks opioid tolerance and increases vulnerability to heroin self-administration in mice. J. Neurosci..

[30] Fowler CD, Lu Q, Johnson PM, Marks MJ, Kenny PJ (2011). Habenular alpha5 nicotinic receptor subunit signalling controls nicotine intake. Nature.

[31] Xi ZX (2011). Brain cannabinoid CB(2) receptors modulate cocaine’s actions in mice. Nat. Neurosci..

[32] Trigo JM (2007). 3,4-methylenedioxymethamphetamine self-administration is abolished in serotonin transporter knockout mice. Biol. Psychiatry.

[33] Han DD, Gu HH (2006). Comparison of the monoamine transporters from human and mouse in their sensitivities to psychostimulant drugs. BMC Pharmacol..

[34] Beckstead MJ, Grandy DK, Wickman K, Williams JT (2004). Vesicular dopamine release elicits an inhibitory postsynaptic current in midbrain dopamine neurons. Neuron.

[35] Groves PM, Wilson CJ, Young SJ, Rebec GV (1975). Self-inhibition by dopaminergic neurons. Science.

[36] Chen NH, Reith ME (1994). Autoregulation and monoamine interactions in the ventral tegmental area in the absence and presence of cocaine: a microdialysis study in freely moving rats. J. Pharmacol. Exp. Ther..

[37] Lacey MG, Mercuri NB, North RA (1990). Actions of cocaine on rat dopaminergic neurones in vitro. Br. J. Pharmacol..

[38] Einhorn LC, Johansen PA, White FJ (1988). Electrophysiological effects of cocaine in the mesoaccumbens dopamine system: studies in the ventral tegmental area. J. Neurosci..

[39] Blier P, Pineyro G, el Mansari M, Bergeron R, de Montigny C (1998). Role of somatodendritic 5-HT autoreceptors in modulating 5-HT neurotransmission. Ann. N. Y. Acad. Sci..

[40] Brown MT, Henny P, Bolam JP, Magill PJ (2009). Activity of neurochemically heterogeneous dopaminergic neurons in the substantia nigra during spontaneous and driven changes in brain state. J. Neurosci..

[41] Tao R, Auerbach SB (1994). Anesthetics block morphine-induced increases in serotonin release in rat CNS. Synapse.

[42] Trulson ME, Trulson VM (1983). Chloral hydrate anesthesia alters the responsiveness of dorsal raphe neurons to psychoactive drugs. Life Sci..

[43] Jolas T, Aghajanian GK (1997). Opioids suppress spontaneous and NMDA-induced inhibitory postsynaptic currents in the dorsal raphe nucleus of the rat in vitro. Brain Res..

[44] Stiller CO, Bergquist J, Beck O, Ekman R, Brodin E (1996). Local administration of morphine decreases the extracellular level of GABA in the periaqueductal gray matter of freely moving rats. Neurosci. Lett..

[45] Voisin AN (2016). Axonal Segregation and Role of the Vesicular Glutamate Transporter VGLUT3 in Serotonin Neurons. Front. Neuroanat..

[46] Pidoplichko VI, DeBiasi M, Williams JT, Dani JA (1997). Nicotine activates and desensitizes midbrain dopamine neurons. Nature.

[47] Mansvelder HD, McGehee DS (2000). Long-term potentiation of excitatory inputs to brain reward areas by nicotine. Neuron.

[48] Koranda JL (2014). Nicotinic receptors regulate the dynamic range of dopamine release in vivo. J. Neurophysiol..

[49] Eddine R (2015). A concurrent excitation and inhibition of dopaminergic subpopulations in response to nicotine. Sci. Rep..

[50] Erhardt S, Schwieler L, Engberg G (2002). Excitatory and inhibitory responses of dopamine neurons in the ventral tegmental area to nicotine. Synapse.

[51] Tolu S (2013). Co-activation of VTA DA and GABA neurons mediates nicotine reinforcement. Mol. Psychiatry.

[52] Tan KR (2012). GABA neurons of the VTA drive conditioned place aversion. Neuron.

[53] van Zessen R, Phillips JL, Budygin EA, Stuber GD (2012). Activation of VTA GABA neurons disrupts reward consumption. Neuron.

[54] Ribeiro EB, Bettiker RL, Bogdanov M, Wurtman RJ (1993). Effects of systemic nicotine on serotonin release in rat brain. Brain Res..

[55] Luscher C, Jan LY, Stoffel M, Malenka RC, Nicoll RA (1997). G protein-coupled inwardly rectifying K + channels (GIRKs) mediate postsynaptic but not presynaptic transmitter actions in hippocampal neurons. Neuron.

[56] Threlfell S (2012). Striatal dopamine release is triggered by synchronized activity in cholinergic interneurons. Neuron.

[57] Colgan LA, Cavolo SL, Commons KG, Levitan ES (2012). Action potential-independent and pharmacologically unique vesicular serotonin release from dendrites. J. Neurosci..

[58] Mejias-Aponte CA, Ye C, Bonci A, Kiyatkin EA, Morales M (2015). A subpopulation of neurochemically-identified ventral tegmental area dopamine neurons is excited by intravenous cocaine. J. Neurosci..

[59] Kuhar MJ, Ritz MC, Boja JW (1991). The dopamine hypothesis of the reinforcing properties of cocaine. Trends Neurosci..

[60] Morgan MJ (2000). Ecstasy (MDMA): a review of its possible persistent psychological effects. Psychopharmacol. (Berl.).

[61] Aragona BJ (2009). Regional specificity in the real-time development of phasic dopamine transmission patterns during acquisition of a cue-cocaine association in rats. Eur. J. Neurosci..

[62] Willuhn I, Burgeno LM, Groblewski PA, Phillips PE (2014). Excessive cocaine use results from decreased phasic dopamine signaling in the striatum. Nat. Neurosci..

[63] Stuber GD, Hnasko TS, Britt JP, Edwards RH, Bonci A (2010). Dopaminergic terminals in the nucleus accumbens but not the dorsal striatum corelease glutamate. J. Neurosci..

[64] Zhang S (2015). Dopaminergic and glutamatergic microdomains in a subset of rodent mesoaccumbens axons. Nat. Neurosci..

[65] Sell LA (1999). Activation of reward circuitry in human opiate addicts. Eur. J. Neurosci..

[66] Volkow ND (1997). Decreased striatal dopaminergic responsiveness in detoxified cocaine-dependent subjects. Nature.

[67] Parrott AC (2001). Human psychopharmacology of Ecstasy (MDMA): a review of 15 years of empirical research. Hum. Psychopharmacol..

[68] Henningfield JE, Miyasato K, Jasinski DR (1985). Abuse liability and pharmacodynamic characteristics of intravenous and inhaled nicotine. J. Pharmacol. Exp. Ther..

[69] Badiani A, Belin D, Epstein D, Calu D, Shaham Y (2011). Opiate versus psychostimulant addiction: the differences do matter. Nat. Rev. Neurosci..

[70] Russo SJ (2010). The addicted synapse: mechanisms of synaptic and structural plasticity in nucleus accumbens. Trends Neurosci..

[71] Caprioli D (2009). Ambience and drug choice: cocaine- and heroin-taking as a function of environmental context in humans and rats. Biol. Psychiatry.

[72] Fowler CD, Kenny PJ (2011). Intravenous nicotine self-administration and cue-induced reinstatement in mice: effects of nicotine dose, rate of drug infusion and prior instrumental training. Neuropharmacology.

[73] Wang D. *et al*. Learning shapes the aversion and reward responses of lateral habenula neurons. *eLife* **6**, e23045 (2017). 10.7554/eLife.23045 PMC5469615 28561735

