# Supplementary material for: Response dynamics of midbrain dopamine neurons and serotonin neurons to heroin, nicotine, cocaine, and MDMA
Source: Cell Discov. 2018 Nov 6;4:60. doi: 10.1038/s41421-018-0060-z (PMC6218454; doi:10.1038/s41421-018-0060-z)
Supplement: Supplementary file 1 — Supplementary Information [file 41421_2018_60_MOESM1_ESM.pdf]

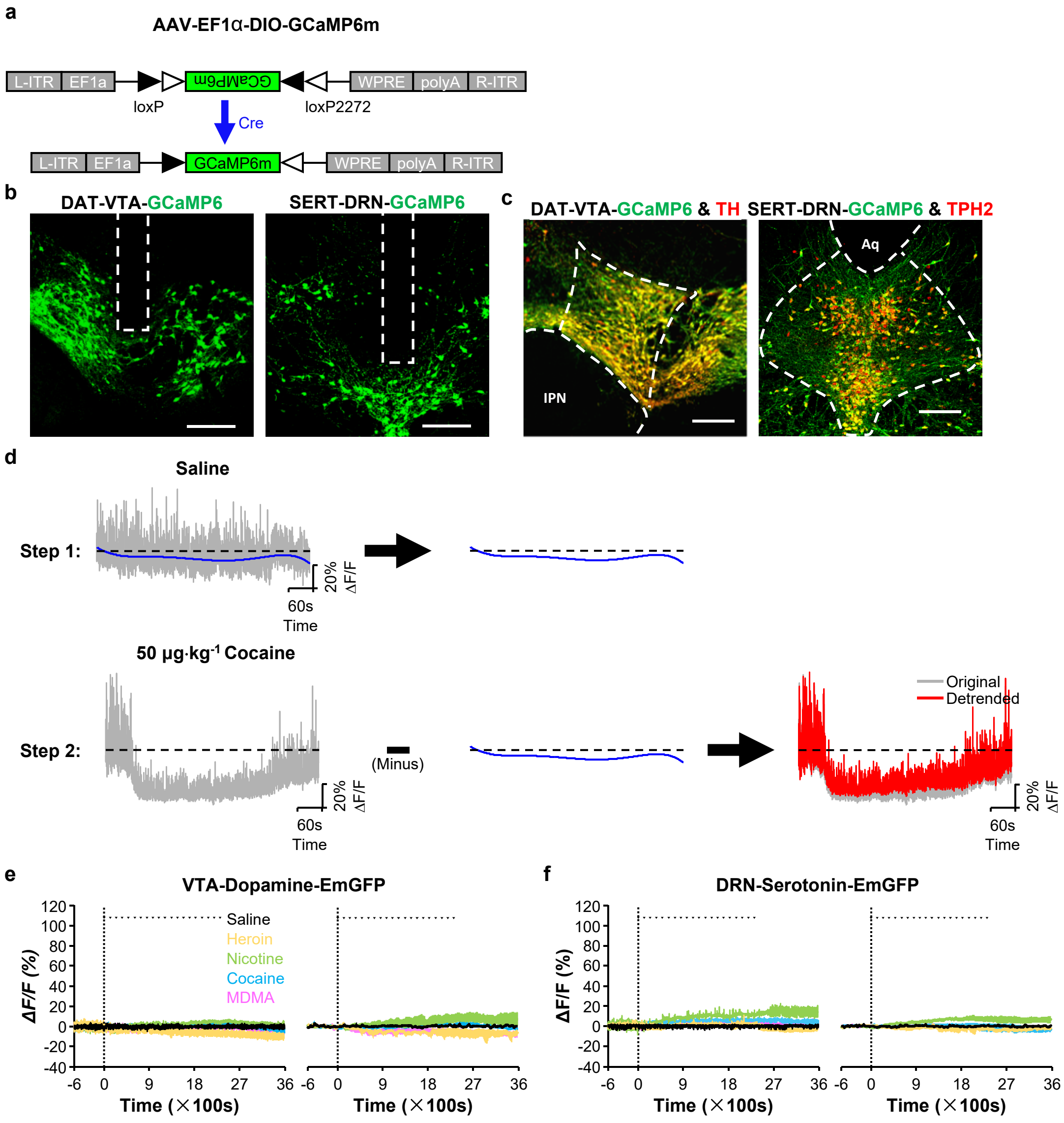

Supplementary Figure S1. The method of fiber photometry and correction of Ca^{2+} signals. a Confocal images of example coronal sections from a DAT-VTA-GCaMP6 mouse (left) and a Sert-DRN-GCaMP6 mouse (right), illustrating GCaMP6m expression and fiber placement in the VTA and the DRN, respectively. Scale bar, 200 μm. **b, c** Confocal images showing GCaMP6m expression (green) in TH-positive (red) dopamine neurons in the VTA of a DAT-VTA-GCaMP6 mouse (**b**) and that in TPH2-positive serotonin neurons in the DRN of a Sert-DRN-GCaMP6 mouse (**c**). **d** The method of correcting GCaMP6m signals for photobleaching. First, we extracted the trending curve of GCaMP fluorescence change upon saline treatment. Second, the GCaMP fluorescence change associated with drug administration was detrended by subtracting the trending curve associated with saline infusion from the raw curve. **e, f** EmGFP fluorescence recorded from dopamine neurons [**e**; left, raw traces of EmGFP fluorescence; right, average traces of EmGFP fluorescence (n = 4)] and serotonin neurons [**f**; left, raw traces of EmGFP fluorescence; right, average traces of EmGFP fluorescence (n = 4)] , showing the lack of clear fluorescence change following drug delivery. Error bars indicate SEM.

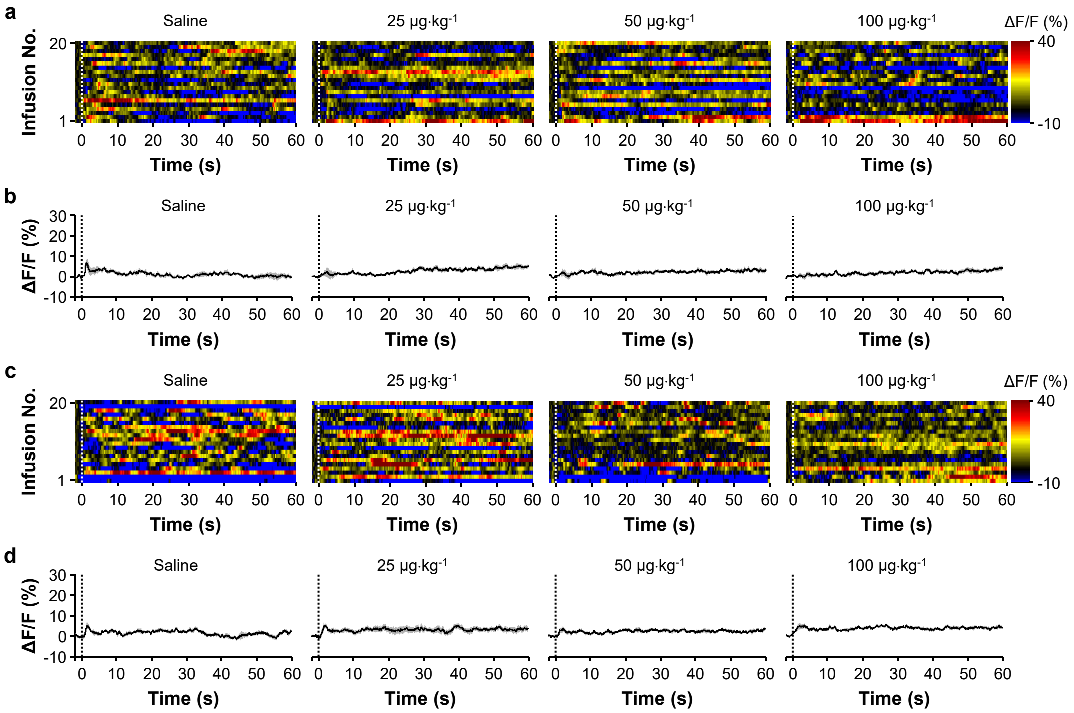

Supplementary Figure S2. Ca^{2+} signals of dopamine and serotonin neurons in response to individual heroin infusion. a Representative heatmap representations of Ca^{2+} signals recorded from dopamine neurons in response to 20 consecutive infusions of heroin at the indicated doses. The white dash line indicates the onset of each infusion. **b** Average Ca^{2+} signals of dopamine neurons aligned to the onset of individual heroin infusions at the indicated doses (n = 6 mice). The black dash line indicates the onset of each infusion. **c** Representative heatmap representations of Ca^{2+} signals recorded from serotonin neurons in response to 20 individual infusions of heroin at the indicated doses. **d** Average Ca^{2+} signals recorded of serotonin neurons aligned to the onset of each heroin infusion (n = 7 mice).

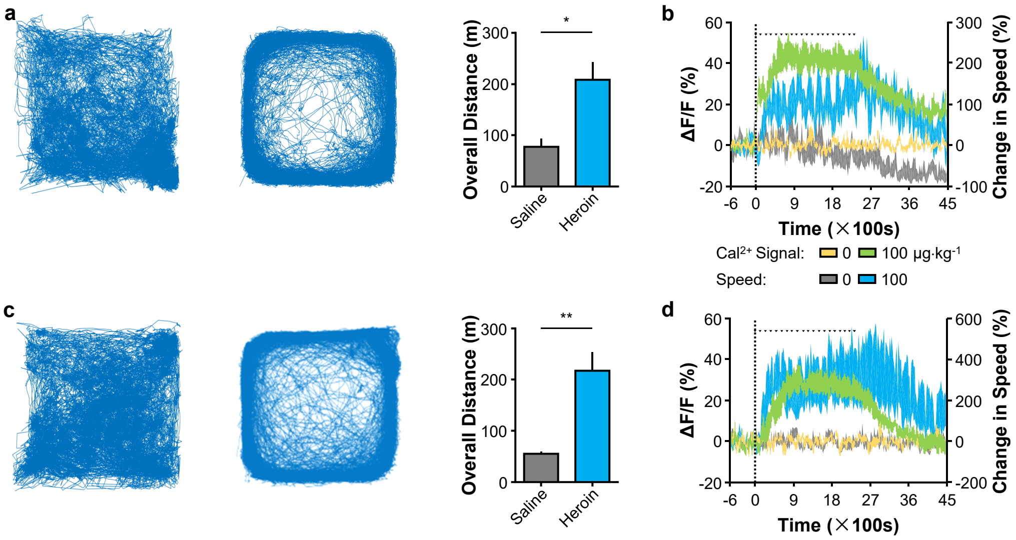

Supplementary Figure S3. Behavioral correlates of heroin-induced effects on Ca^{2+} signals of dopamine neurons and serotonin neurons. a Representative locomotor trajectories of a dopamine mouse responding to saline (left panel) and 100 μg·kg^{-1} heroin (middle panel), and overall travelling distance challenged by saline and 100 μg·kg^{-1} heroin (right panel; n = 6 dopamine mice; paired t-test, p = 0.0141). **b** Association of heroin-induced changes in Ca^{2+} signals of dopamine neurons with travelling speed (n = 6 dopamine mice). **c** Representative locomotor trajectories of a serotonin mouse responding to saline (left panel) and 100 μg·kg^{-1} heroin (middle panel), and overall travelling distance challenged by saline and 100 μg·kg^{-1} heroin (right panel; n = 7 serotonin mice; paired t-test, p = 0.0054). **d** Association of heroin-induced changes in Ca^{2+} signals of serotonin neurons with travelling speed(n = 7 serotonin mice). Error bars indicate SEM (**b** and **d**). * $p < 0.05$; ** $p < 0.01$; *** $p < 0.001$; ns, not significant.

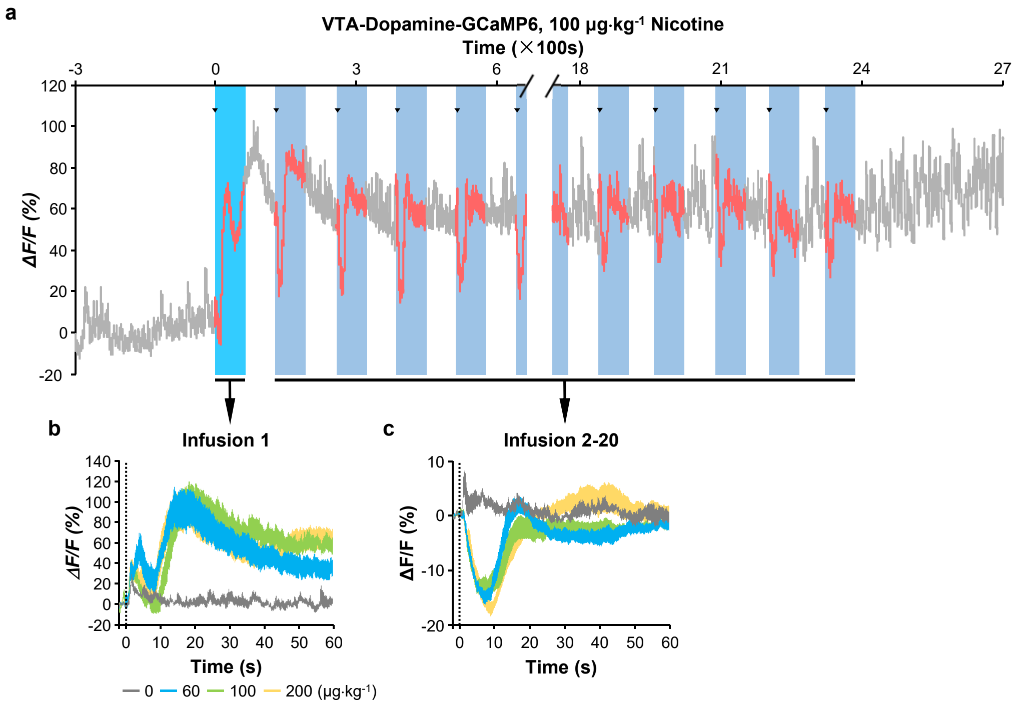

Supplementary Figure S4. Nicotine phasically modulates the activity of VTA dopamine neurons with a biphasic pattern. **a** Zoomed-in view of Ca^{2+} signals from dopamine neurons illustrating the biphasic response pattern of dopamine neurons following each nicotine infusion. **b** Average plots of fluorescence intensity, aligned to the onset of the first infusion, revealing the peri-event activities of dopamine neurons in response to the first nicotine infusion (n = 6 dopamine mice). **c** Plot of mean fluorescence intensity, aligned to the start of each infusion, showing the peri-event activities of dopamine neurons in response to nicotine infusions 2-20 (n = 6 dopamine mice).

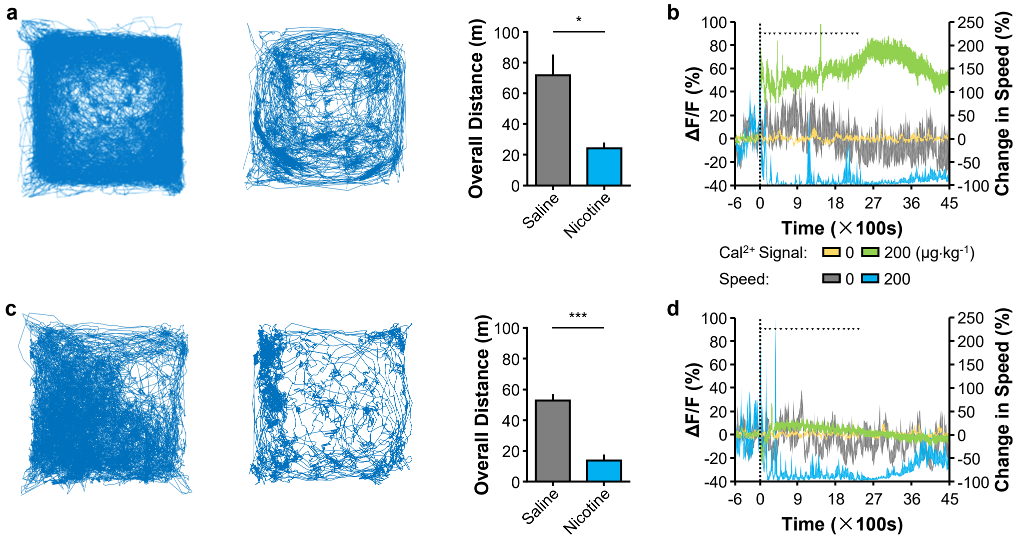

Supplementary Figure S5. Behavioral correlates of nicotine-induced effects on Ca^{2+} signals of dopamine neurons and serotonin neurons. a Locomotor trajectories of a representative dopamine mouse responding to saline (left panel) and 200 μg·kg^{-1} nicotine (middle panel), and overall travelling distance challenged by saline and μg·kg^{-1} nicotine (right panel; n = 6 dopamine mice; paired t-test, p = 0.0281). **b** Association of nicotine-induced changes in Ca^{2+} signals of dopamine neurons with travelling speed (n = 6 dopamine mice). **c** Locomotor trajectories of a representative serotonin mouse responding to saline (left panel) and 200 μg·kg^{-1} nicotine (right panel), and overall travelling distance challenged by saline and 200 μg·kg^{-1} nicotine (right panel; n = 7 serotonin mice; paired t-test, p = 0.0002). **d** Association of nicotine-induced changes in Ca^{2+} signals of serotonin neurons with travelling speed (n = 7 serotonin mice). Error bars indicate SEM (**b** and **d**). * $p < 0.05$; ** $p < 0.01$; *** $p < 0.001$; ns, not significant.

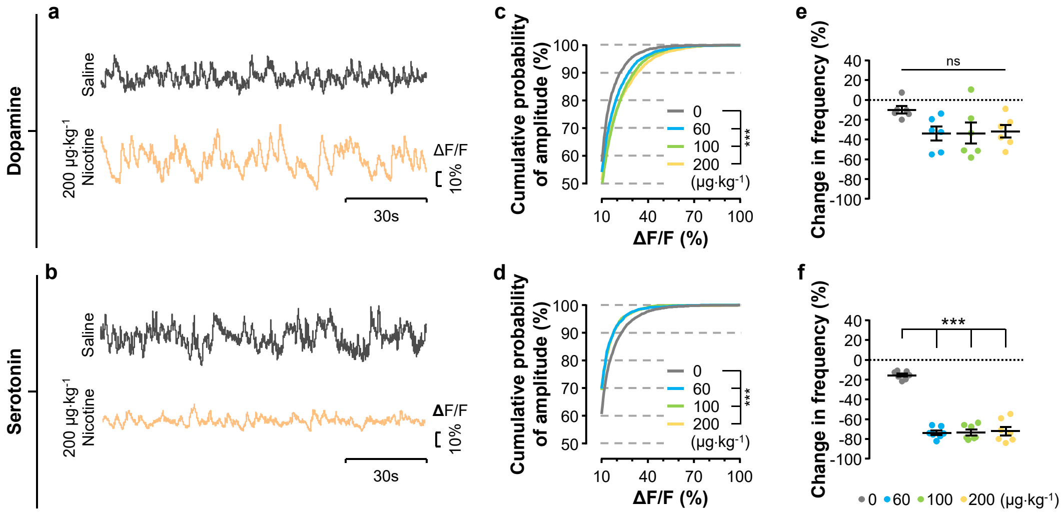

Supplementary Figure S6. The effects of nicotine on Ca^{2+} transients of dopamine neurons and serotonin neurons. a, **b** Representative traces of GCaMP6m signals from dopamine neurons (**a**) and serotonin neurons (**b**) during nicotine infusion. **c, d** Cumulative probability distribution of Ca^{2+} transient amplitudes for dopamine neurons (**c**) and serotonin neurons (**d**) following the deliveries of nicotine at different doses (n = 6 dopamine mice; n = 7 serotonin mice; K-S test). **e, f** The effect of nicotine infusions at different doses on change in the frequency of Ca^{2+} transients (calcium events per 10 min) of dopamine neurons (**e**) and serotonin neurons (**f**) before versus during nicotine application [n = 6 dopamine mice, $F(3, 20) = 2.504$, $p = 0.0885$, one-way ANOVA with Tukey's post-hoc test; n = 7 serotonin mice, $F(3, 24) = 101.3$, $p < 0.0001$, one-way ANOVA with Tukey's post-hoc test]. Error bars indicate SEM (**e**, **f**). * $p < 0.05$; ** $p < 0.01$; *** $p < 0.001$; ns, not significant.

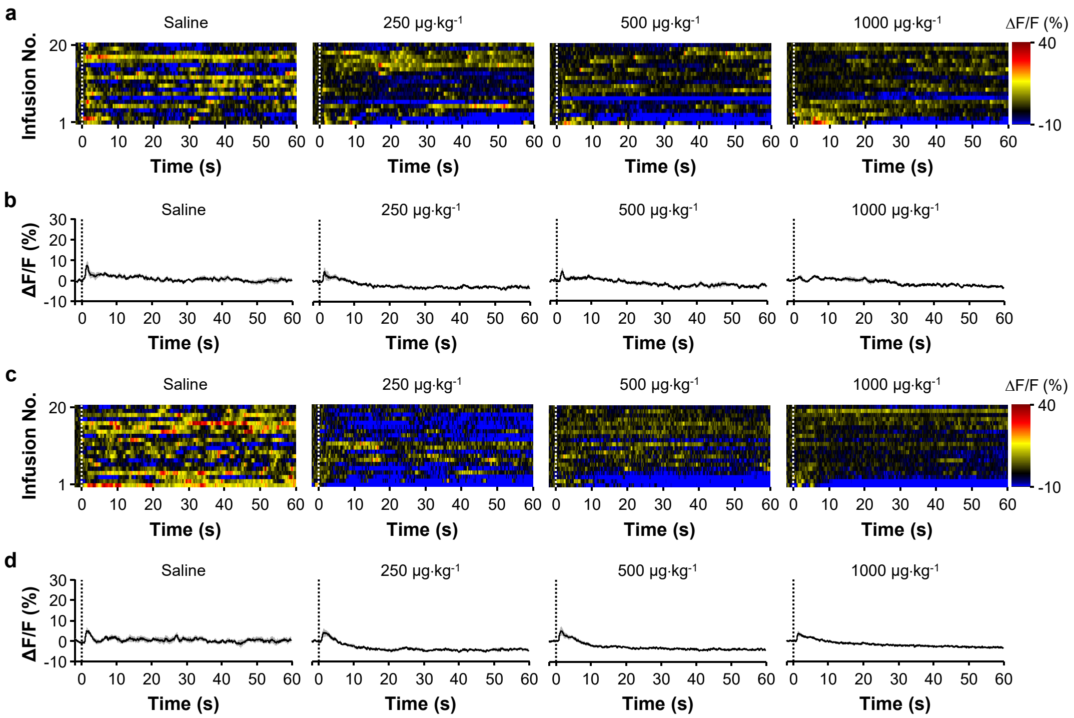

Supplementary Figure S7. Ca^{2+} signals of dopamine and serotonin neurons in response to individual cocaine infusions. a Representative heatmap representations of Ca^{2+} signals recorded from dopamine neurons in response to 20 consecutive infusions of cocaine at the indicated doses. The white dash line indicates the onset of each infusion. **b** Average Ca^{2+} signals of dopamine neurons aligned to the onset of individual cocaine infusions at the indicated doses (n = 7 mice). The black dash line indicates the onset of each infusion. **c** Representative heatmap representations of Ca^{2+} signals recorded from serotonin neurons in response to 20 infusions of cocaine at the indicated doses. **d** Average Ca^{2+} signals recorded of serotonin neurons aligned to the onset of individual cocaine infusions (n = 7 mice).

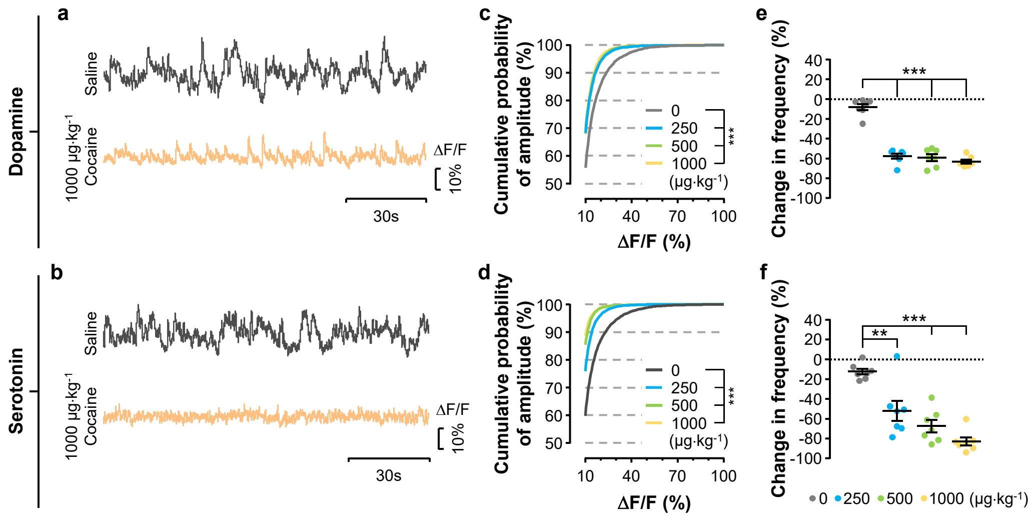

Supplementary Figure S8. The effects of cocaine on Ca^{2+} transients of dopamine neurons and serotonin neurons. a, **b** Zoom-in view of Ca^{2+} signals for dopamine neurons (**a**) and serotonin neurons (**b**) during cocaine application. **c**, **d** Cumulative probability distribution of Ca^{2+} transient amplitudes of dopamine neurons (**c**) and serotonin neurons (**d**) during cocaine application (dopamine, n = 7; serotonin, n = 7; K-S test). **e**, **f** The effect of cocaine administrations at different doses on change in the frequency of Ca^{2+} transients (calcium events per 10 min) of dopamine neurons (**e**) and serotonin neurons (**f**) before versus during cocaine application [n = 7 dopamine mice, $F(3, 24) = 86.13$, $p < 0.0001$, one-way ANOVA with Tukey's post-hoc test; n = 7 serotonin mice, $F(3, 24) = 21.91$, $p < 0.0001$, one-way ANOVA with Tukey's post-hoc test]. Error bars indicate the standard error of the mean (SEM) (**e** and **f**). * $p < 0.05$; ** $p < 0.01$; *** $p < 0.001$; ns, not significant.

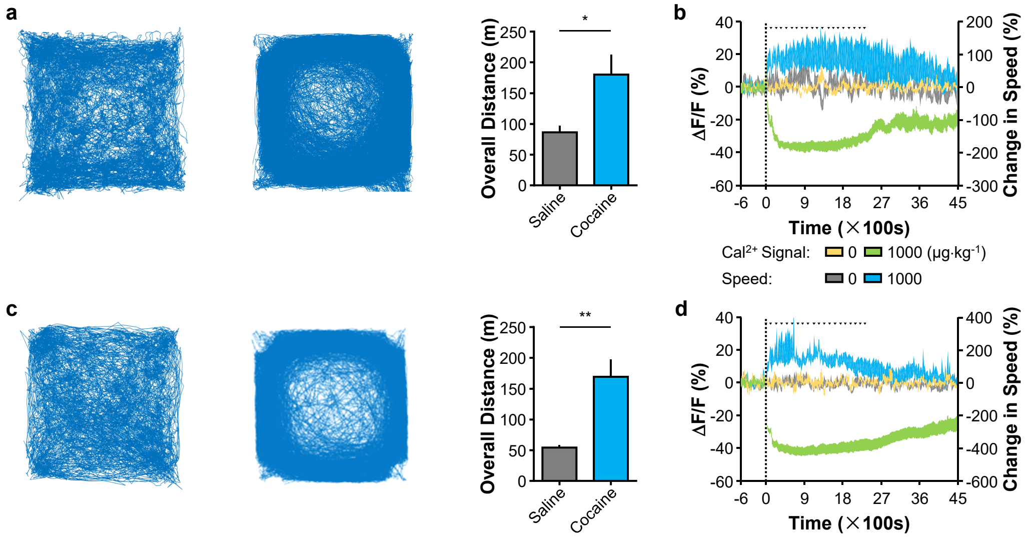

Supplementary Figure S9. Behavioral correlates of cocaine-induced effects on Ca^{2+} signals of dopamine neurons and serotonin neurons. a Representative locomotor trajectories of a dopamine mouse responding to saline (left panel) and 1000 $\mu g \cdot kg^{-1}$ cocaine (middle panel), and overall travelling distance challenged by saline and 1000 $\mu g \cdot kg^{-1}$ cocaine (right panel; n = 7 dopamine mice; paired t-test, p = 0.0447). **b** Association of cocaine-induced changes in Ca^{2+} signals of dopamine neurons with travelling speed (n = 7 dopamine mice). **c** Representative locomotor trajectories of a serotonin mouse responding to saline (left panel) and 1000 $\mu g \cdot kg^{-1}$ cocaine (middle panel), and overall travelling distance challenged by saline and 1000 $\mu g \cdot kg^{-1}$ cocaine (right panel; n = 7 serotonin mice; paired t-test, p = 0.0059). **d** Association of cocaine-induced changes in Ca^{2+} signals of serotonin neurons with travelling speed (n = 7 serotonin mice). Error bars indicate the standard error of the mean (SEM) (**b** and **d**). * $p < 0.05$; ** $p < 0.01$; *** $p < 0.001$; ns, not significant.

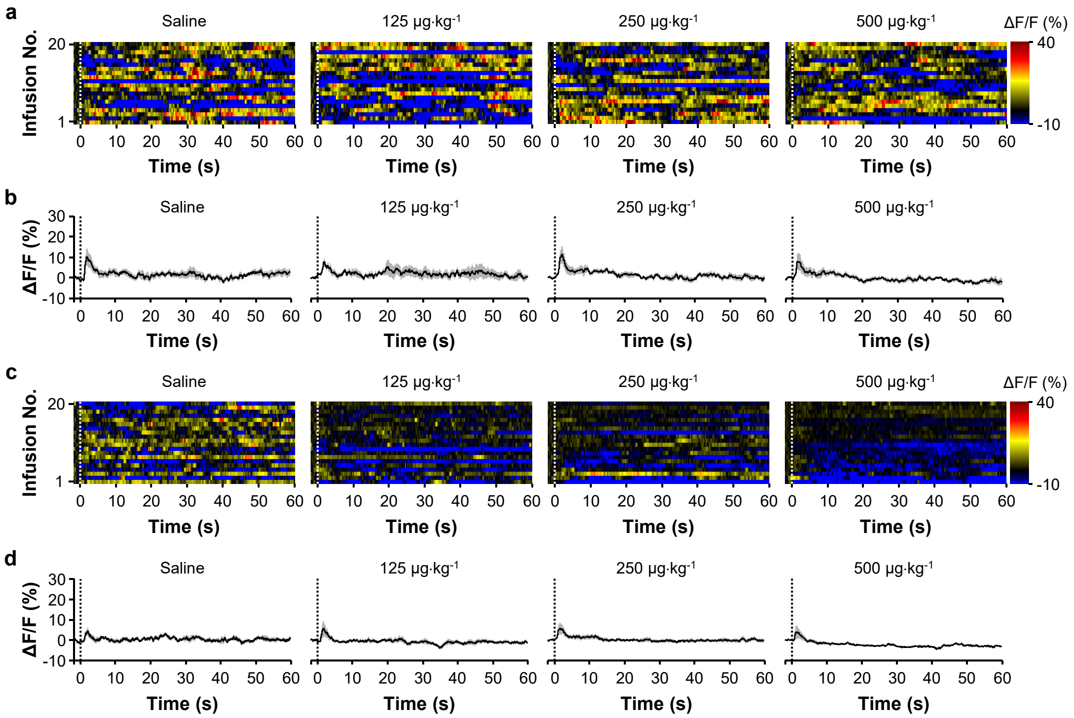

Supplementary Figure S10. Ca^{2+} signals of dopamine and serotonin neurons in response to individual MDMA infusions. a Representative heatmap representations of Ca^{2+} signals recorded from dopamine neurons in response to 20 infusions of MDMA at the indicated doses. The white dash line indicates the onset of each infusion. **b** Average Ca^{2+} signals of dopamine neurons aligned to the onset of individual MDMA infusions at the indicated doses (n = 6 mice). The black dash line indicates the onset of each infusion. **c** Representative heatmap representations of Ca^{2+} signals recorded from serotonin neurons in response to 20 infusions of MDMA at the indicated doses. **d** Average Ca^{2+} signals recorded of serotonin neurons aligned to the onset of individual MDMA infusions (n = 7 mice).

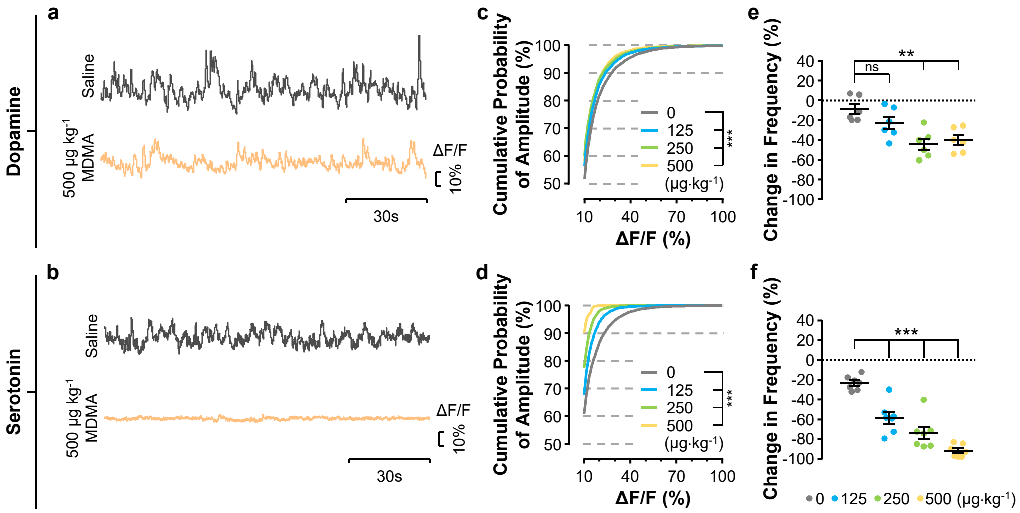

Supplementary Figure S11. The effects of MDMA on Ca^{2+} transients of dopamine neurons and serotonin neurons. a, **b** Zoom-in view of Ca^{2+} signals for dopamine neurons (**a**) and serotonin neurons (**b**) during MDMA application. **c**, **d** Cumulative probability distribution of Ca^{2+} transient amplitudes of dopamine neurons (**c**) and serotonin neurons (**d**) during MDMA application (n = 6 dopamine mice; n = 7 serotonin mice; K-S test). **e**, **f** The effect of MDMA infusions at different doses on change in the frequency of Ca^{2+} transients (calcium events per 10 min) of dopamine neurons (**e**) and serotonin neurons (**f**) before versus during MDMA application [n = 6 dopamine mice, $F(3, 20) = 8.602$, $p = 0.0007$, one-way ANOVA with Tukey's post-hoc test; n = 7 serotonin mice, $F(3, 24) = 39.10$, $p < 0.0001$, one-way ANOVA with Tukey's post-hoc test]. Error bars indicate SEM (**e** and **f**). * $p < 0.05$; ** $p < 0.01$; *** $p < 0.001$; ns, not significant.

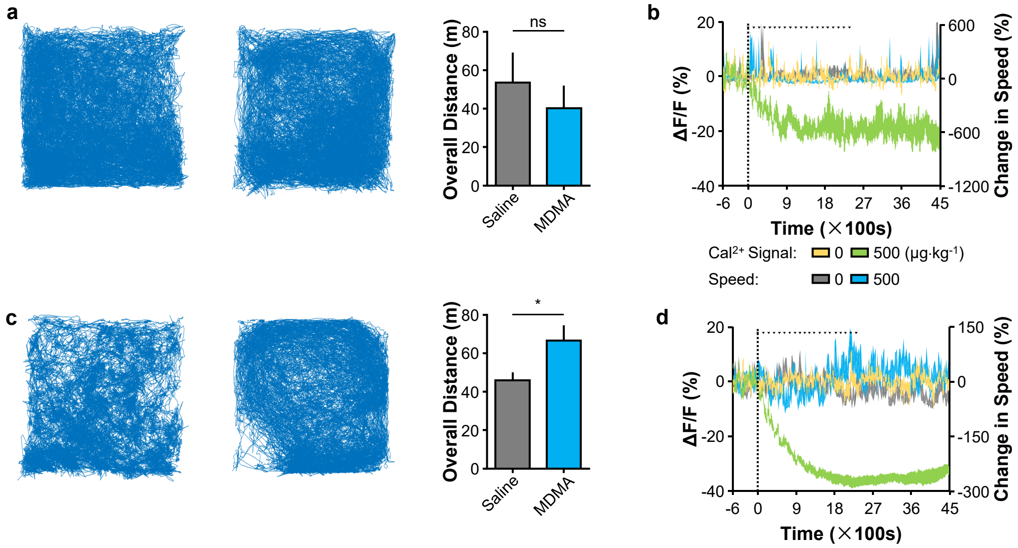

Supplementary Figure S12. Behavioral correlates of MDMA-induced effects on Ca^{2+} signals of dopamine neurons and serotonin neurons. a Representative locomotor trajectories of a representative dopamine mouse responding to saline (left panel) and 500 μg·kg^{-1} MDMA (middle panel), and overall travelling distance challenged by saline and 500 μg·kg^{-1} MDMA (right panel; n = 6 dopamine mice; paired t-test, p = 0.3367). **b** Association of MDMA-induced changes in Ca^{2+} signals of dopamine neurons with travelling speed (n = 6 dopamine mice). **c** Representative locomotor trajectories of a serotonin mouse responding to saline (left panel) and 500 μg·kg^{-1} MDMA (middle panel), and overall travelling distance challenged by saline and 500 μg·kg^{-1} MDMA (right panel; n = 7 serotonin mice; paired t-test, p = 0.0227). **d** Association of MDMA-induced changes in Ca^{2+} signals of serotonin neurons with travelling speed (n = 7 serotonin mice). Error bars indicate SEM (**c** and **d**). * $p < 0.05$; ** $p < 0.01$; *** $p < 0.001$; ns, not significant.

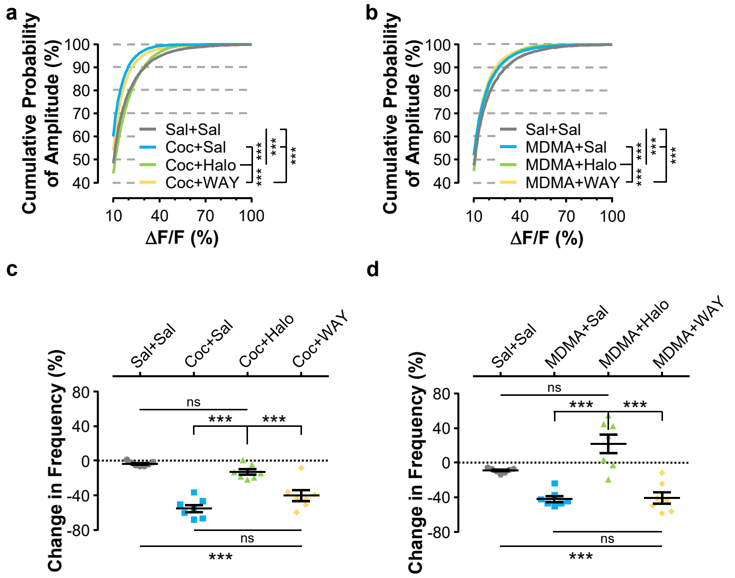

Supplementary Figure S13. Cocaine and MDMA inhibit dopamine neurons via DRD2 receptor activity. **a, b** The effect of haloperidol and WAY100635 on the cumulative probability distribution of Ca^{2+} transient amplitudes for dopamine neurons following the infusion of cocaine (**a**) or MDMA (**b**) (n = 7 for each test group; K-S test). **c, d** The effect of haloperidol and WAY100635 on change in Ca^{2+} transient frequencies for dopamine neurons (calcium events per 10 min) before versus during cocaine application illustrated in Figure **c** [n = 7 dopamine mice, $F(3, 24) = 34.74$, $p < 0.0001$, one-way ANOVA with Tukey's post-hoc test) or MDMA application illustrated in Figure **d** (n = 7 dopamine mice, $F(3, 24) = 21.88$, $p < 0.0001$, one-way ANOVA with Tukey's post-hoc test]. Error bars indicate SEM (**c**, **d**). * $p < 0.05$; ** $p < 0.01$; *** $p < 0.001$; ns, not significant.

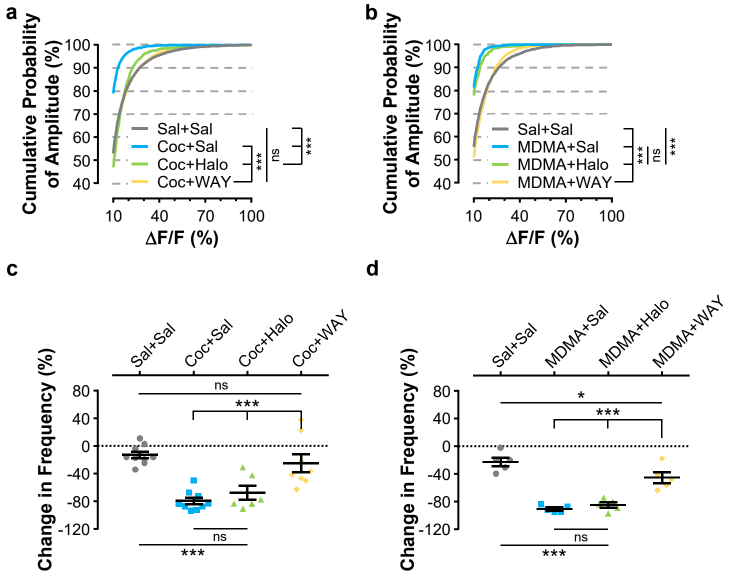

Supplementary Figure S14. The activity of HTR1A receptors contribute to cocaine- and MDMA-induced inhibition of serotonin neurons. a, b The effect of haloperidol and WAY100635 on the cumulative probability distribution of Ca^{2+} transient amplitudes for serotonin neurons following the infusion of cocaine (**a**) or MDMA (**b**) (K-S test employed for statistic analysis of each test group). **c, d** The effect of haloperidol and WAY100635 on change in Ca^{2+} transient frequencies for serotonin neurons (calcium events per 10 min) before versus during cocaine application illustrated in Figure **c** [F(3, 28) = 15.90, p < 0.0001, one-way ANOVA with Tukey's post-hoc test] or MDMA application illustrated in Figure **d** [F(3, 16) = 34.51, p < 0.0001, one-way ANOVA with Tukey's post-hoc]. Error bars indicate SEM (**c**, **d**). * *p* < 0.05; ** *p* < 0.01; *** *p* < 0.001; ns, not significant. n = 9 serotonin mice for Sal+Sal and Sal+Coc, n = 6 for Halo+Coc, n = 8 for WAY+Coc (**a**, **c**); n = 5 for Sal+Sal, Sal+MDMA, Halo+MDMA, and WAY+MDMA (**b**, **d**).

Supplementary Table S1

	Mice ID	Drug Treatment Order							
DAT-Cre Mice	DAT #01	Saline	Cocaine	4 days rest	Heroin			4 days rest	Nicotine
	DAT #02	Saline	Cocaine	4 days rest	Heroin			4 days rest	Nicotine
	DAT #03	Saline	Cocaine	4 days rest	Heroin			4 days rest	Nicotine
	DAT #04	Saline	Cocaine	4 days rest	Heroin			4 days rest	Nicotine
	DAT #05	Saline	Cocaine	4 days rest	Heroin			4 days rest	Nicotine
	DAT #06	Saline	Cocaine	4 days rest	Heroin			4 days rest	Nicotine
	DAT #07	Saline	Cocaine						
	DAT #08	Saline					MDMA		
	DAT #09	Saline					MDMA		
	DAT #10	Saline					MDMA		
	DAT #11	Saline					MDMA		
	DAT #12	Saline					MDMA		
	DAT #13	Saline					MDMA		

	Mice ID	Drug Treatment Order							
SERT-Cre Mice	SERT #01	Saline	Cocaine	4 days rest	Heroin	4 days rest	MDMA	4 days rest	Nicotine
	SERT #02	Saline	Cocaine	4 days rest	Heroin			4 days rest	Nicotine
	SERT #03	Saline	Cocaine	4 days rest	Heroin				
	SERT #04	Saline	Cocaine	4 days rest	Heroin				
	SERT #05	Saline	Cocaine					4 days rest	Nicotine
	SERT #06	Saline	Cocaine						
	SERT #07	Saline	Cocaine						
	SERT #08	Saline			Heroin	4 days rest	MDMA	4 days rest	Nicotine
	SERT #09	Saline			Heroin			4 days rest	Nicotine
	SERT #10	Saline			Heroin				
	SERT #11	Saline					MDMA	4 days rest	Nicotine
	SERT #12	Saline					MDMA	4 days rest	Nicotine
	SERT #13	Saline					MDMA		
	SERT #14	Saline					MDMA		
	SERT #15	Saline					MDMA		
